# Towards identification of postharvest fruit quality transcriptomic markers in *Malus domestica*

**DOI:** 10.1371/journal.pone.0297015

**Published:** 2024-03-06

**Authors:** John A. Hadish, Heidi L. Hargarten, Huiting Zhang, James P. Mattheis, Loren A. Honaas, Stephen P. Ficklin

**Affiliations:** 1 Molecular Plant Science Department, Washington State University, Pullman, Washington, United States of America; 2 Department of Horticulture, Washington State University, Pullman, Washington, United States of America; 3 USDA Agricultural Research Service Physiology and Pathology of Tree Fruits Research, Wenatchee, Washington, United States of America; Nuclear Science and Technology Research Institute, ISLAMIC REPUBLIC OF IRAN

## Abstract

Gene expression is highly impacted by the environment and can be reflective of past events that affected developmental processes. It is therefore expected that gene expression can serve as a signal of a current or future phenotypic traits. In this paper we identify sets of genes, which we call Prognostic Transcriptomic Biomarkers (PTBs), that can predict firmness in *Malus domestica* (apple) fruits. In apples, all individuals of a cultivar are clones, and differences in fruit quality are due to the environment. The apples transcriptome responds to these differences in environment, which makes PTBs an attractive predictor of future fruit quality. PTBs have the potential to enhance supply chain efficiency, reduce crop loss, and provide higher and more consistent quality for consumers. However, several questions must be addressed. In this paper we answer the question of which of two common modeling approaches, Random Forest or ElasticNet, outperforms the other. We answer if PTBs with few genes are efficient at predicting traits. This is important because we need few genes to perform qPCR, and we answer the question if qPCR is a cost-effective assay as input for PTBs modeled using high-throughput RNA-seq. To do this, we conducted a pilot study using fruit texture in the ‘Gala’ variety of apples across several postharvest storage regiments. Fruit texture in ‘Gala’ apples is highly controllable by post-harvest treatments and is therefore a good candidate to explore the use of PTBs. We find that the RandomForest model is more consistent than an ElasticNet model and is predictive of firmness (r2 = 0.78) with as few as 15 genes. We also show that qPCR is reasonably consistent with RNA-seq in a follow up experiment. Results are promising for PTBs, yet more work is needed to ensure that PTBs are robust across various environmental conditions and storage treatments.

## 1 Introduction

Apples (*Malus domestica*) are one of the world’s most consumed fruits, with over 4.8 billion kilograms produced annually in the United States [1]. Advancements in breeding, orchard management, and postharvest storage technologies have made it possible to store apples for up to a year after harvest [2]. Despite this, producers and packing houses cull millions of kilograms of apples annually due to losses of fruit quality (e.g. loss of firmness and acid), including physiological disorders such as internal browning, bitter pit, superficial scald, and watercore [2–5]. The propensity for losses in quality may not be apparent at harvest and instead develop after apples have been in storage for several months.

Improved predictions about the risk for losses in apple fruit quality could enhance efficiency throughout the supply chain, from the field to the consumer table. For example, apples at relatively high risk for losses in quality could be marketed first, reducing storage costs, and increasing pack-out among fruit lots. The current apple management toolkit is largely composed of physiological indices including starch clearing [6], fruit firmness [7], peel color [8], and acid and sugar content [9]. However, these methods are often insufficient to estimate risk for losses in quality; indeed these limitations certainly play a role in the diversion of billions of kilograms of apple fruit from the fresh fruit market [10].

Prognostic biomarkers are a potential alternative to physiological measurements. These biomarkers consist of one or more biomolecules which can be related to a final outcome. It is typical for apple varieties to be clonally propagated, creating an orchard composed of genetically identical individuals which ensures the production environment yields consistent, cultivar-specific fruit qualities. Because apple cultivars are clones of one another it stands to reason that differences in gene expression at harvest and after postharvest storage are due to environmental effects (e.g., climate, soil, disease pressures and management practices) as trees are effectively genetically identical. The apple fruit transcriptome can respond early and rapidly to changes in production and postharvest environments and could therefore be indicative of future fruit quality. Previously, the term prognostic transcriptomic biomarker (PTB) has been used as describing one or more genes whose expression profile is associated with the occurrence of a phenotypic trait [11]. In the case of apple production, a PTB would be associated with a fruit quality metric, and changes thereof, during the postharvest period. Researchers can identify useful PTBs from RNA-seq data using statistical and machine-learning methods that identify expression profiles associated with complex phenotypic traits [12]. These machine-learning methods do not use prior knowledge of molecular pathways and instead rely only on RNA-seq observations. This means that the identified PTBs are not biased by existing molecular knowledge (i.e., gene function), which can be incomplete in non-model organisms like apple where direct evidence of gene function is oftentimes lacking. Indeed, the majority of research using PTBs has been in the context of human medicine where they have been used to predict complex diseases such as cancer [13, 14], heart disease [15], and Alzheimer’s [16]. However, there have been recent investigations into developing PTBs for predicting commercially relevant apple disorders; biomarker tests were launched commercially in 2019 for risk assessment of bitter pit and soft scald in ‘Honeycrisp’ apples [17–19]. Although these particular markers have since been discontinued, interest in this area remains [2]. For example, a recent study sought to develop PTBs to distinguish among harvest times in ‘Royal Gala’ fruit as a method for determining an optimal harvest date [20]. Despite these efforts, multiple questions remain, such as if common modeling methods are appropriate for PTBs; what the minimum number of genes is needed for a model to be predictive; and if targeted technologies such as qPCR can be used as a more cost effective substitute for application of PTBs in a commercial setting.

In this study, we seek to answer these questions by developing preliminary PTBs for predicting firmness in ‘Gala’ apples, a variety particularly susceptible to loss of firmness during storage [21]. We chose firmness as a proof of concept for PTB identification for three reasons: [1] there already exist several candidate genes within the literature related to firmness identified by genomic means which we can use for model assessment [22], [2] firmness can be easily and accurately measured using a penetrometer [7], and [3] firmness is strongly impacted by postharvest treatments [23, 24] making it controllable within our project’s experimental design. Our experiment was designed to track changes in fruit texture across commercially relevant storage regimes. These included refrigeration, controlled atmosphere [25], and the ethylene perception inhibitor 1-Methylcyclopropene (1-MCP) [26], allowing for the identification of PTBs that are robust across different postharvest conditions.

To explore which predictive models are most appropriate, we explore two common methods for association analysis, Elastic Net (EN) [27] and Random Forest (RF) [28] feature selection, for the identification of potential PTBs using a large RNA-seq and fruit quality dataset. We further verify our PTBs using Boruta feature selection and demonstrate that a relatively small set (15 PTBs) could be sufficient for accurate predictions of loss of firmness within our dataset. Finally, we used qPCR to test the robustness of a subset of potential PTBs in a different lot of ‘Gala’ fruit not used for model development to explore the feasibility for qPCR to be used as a lower-cost assay for models built with RNA-seq.

## 2 Materials and methods

### 2.1 Fruit harvest, sorting, and storage

Commercially viable PTBs must be robust to the expected and inherent variation in environmental conditions and microclimates experienced by fruit being brought to commercial packing houses. Thus, ‘Gala’ fruit was harvested from two different locations in two different years. Fruit harvested in Year 1 (2018) was used for RNA-Seq and model development for selecting PTB candidates, while fruit harvested in Year 2 (2019) was used for qPCR validation of the selected PTB candidates. qPCR validation of a subset of candidate PTBs identified by our models was performed to assess whether such putative candidates would be robust to variations in different growing conditions experienced by the fruit. Fruit from both locations/years, either met commercial packing house standards (Year 1), or were of commercially equivalent quality (Year 2).

Year 1 fruit was received in a single bin from a commercial packing house in Quincy, WA on August 21^st^, 2018. Upon arrival at the USDA-ARS Tree Fruit Research Laboratory in Wenatchee, WA, apples were randomly sorted by hand onto pressed fiber fruit trays holding 18 apples each. Trays were then randomly placed in cardboard boxes, with each box containing 3 trays of fruit, and stored in air at 1°C for seven days. This 7d conditioning period is a standard commercial practice [29] used to mitigate negative storage outcomes that can be associated with early application of controlled atmosphere and 1-MCP.

**Fig 1:**
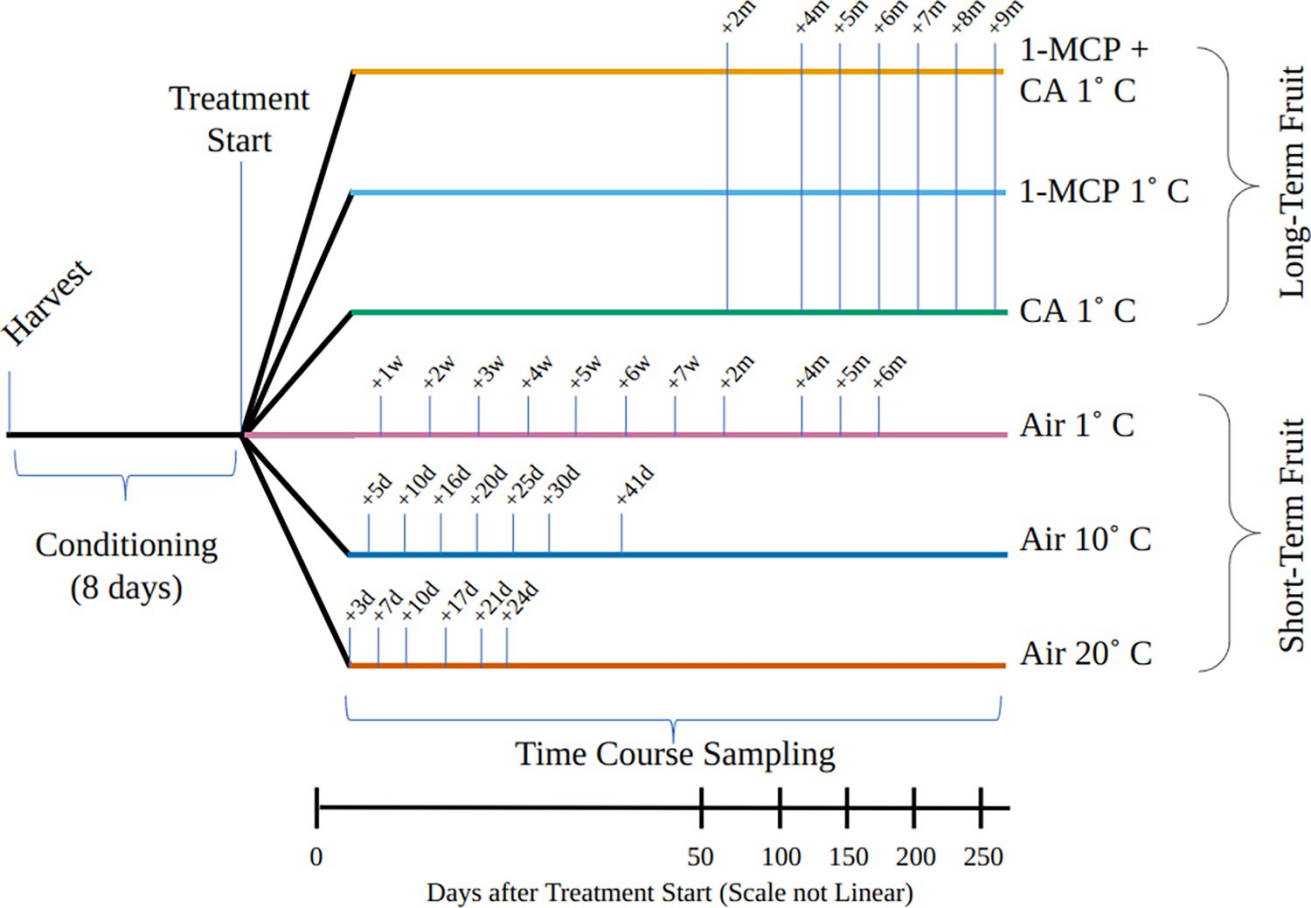
Sampling time points and treatments for 2018 RNA-seq data. Each time point includes the following samples: 3 biological replicates (6 apples per replicate) used for RNA-Seq, a texture analysis of 9 apple fruit at pullout, and a texture analysis of 9 apple fruit after a 7d ripening period (stored in air at 20°C).

After seven days of conditioning, boxes of apples were randomly assigned into one of 6 possible treatment conditions, three “short-term” storage conditions and three “long-term” storage conditions.” The fruit designated for short-term storage were placed in normal air in storage rooms set at either 1°C (A1, n = 396), 10°C (A10, n = 252) or 20°C (A20, n = 216). Fruit designated for long-term storage were divided into three treatment categories (MCP, CA, MCPCA). Two-thirds of these fruits were treated with SmartFresh™ (AgroFresh Solutions, Inc., Philadelphia, PA USA), also known as 1-Methylcyclopropene (1-MCP), overnight. Post treatment, fruit were stored at 1°C in either air (MCP, n = 252) or controlled atmosphere (MCPCA, n = 252; 2% O_2_, 1% CO_2_). The remaining one-third of the fruit not treated with 1-MCP were stored in a controlled atmosphere at 1°C (CA, n = 252; 2% O_2_, 1% CO_2_). 1-MCP was applied at 1°C and in accordance with SmartFresh™ product recommendations. These storage conditions represent commercially equivalent conditions, albeit at a much smaller scale (1,000s of fruit vs 100,000s of fruit). Fruit in the A1 and MCP treatments were stored in the same storage room, and fruit in the CA and MCPCA treatments were stored in separate CA chambers (2 chambers per treatment) within the same storage room. Sampling points and experimental layout are illustrated in Fig 1 and S1 Table in S4 File. For postharvest sampling timelines, condition-relevant time intervals were chosen as short-term fruit was expected to lose firmness faster than long-term fruit. See S1 Table in S4 File for a detailed description of experimental conditions, treatments, and sampling time points for Years 1 and 2.

The following year, a second batch of fruit was harvested for preliminary PTB evaluation. These Year 2 ‘Gala’ apples were harvested from the Washington State University Tree Fruit Research and Extension Center’s Sunrise Research Orchard near Rock Island, WA on August 27th, 2019. Fruit in this research block were managed to be of commercially equivalent fruit quality. These fruits were noted to be larger (at harvest diameter, Welch two sample t-test p = 0.0077, S1A Fig) and slightly more mature (at harvest creep, Welch two sample t-test p = 0.084, S1B Fig) at harvest compared to fruit from Year 1. Year 2 fruit were treated and stored in a similar manner as Year 1, with the A20 experimental group omitted.

### 2.2 Fruit quality, firmness, and tissue collection

For both experiment years, and at each treatment time point, 1 tray of fruit (18 ‘Gala’ apples) was removed from storage and cortex tissue was harvested for RNA extraction. From the 18 fruit three biological replicates were generated, with each replicate comprising of pooled tissue from six apples. Fruit was kept at its storage condition temperature (A20 = 20°C; A10 = 10°C; A1, MCP, MCPCA and CA = 1°C) until the time of tissue harvest. CA fruit was removed from CA prior to tissue harvest (in air for ~ 30 min from removal to completion of tissue collection). Cortex tissue was harvested by first using a vegetable peeler to remove the peel of the apple around the apple’s equator (~ 2 cm wide) to expose cortex tissue. Then, using a knife, an ~ 0.5 cm wide disc was cut from the center of the apple. From this disc, three equal slices of cortex tissue were taken around the circumference of the fruit, omitting core tissue [30]. Slices were coarsely diced and immediately flash-frozen in liquid nitrogen and stored at -80°C. Frozen tissue was ground using a SPEX® Freezer/Mill® 6875 (SPEX®SamplePrep, Metuchen, NJ USA). Ground, frozen tissue was stored at -80°C. Cortex tissue was sampled because the fruit flesh is where fruit texture changes occur and is the actual tissue that is measured by penetrometers used to assess fruit flesh texture.

At each sampling time point, two additional sets of fruit were removed for texture analysis via the Mohr Digi-Test MDT-2 Penetrometer (MOHR Test and Measurement LLC, Richland, WA USA) using the standard 11 mm probe for apple fruit. The first set of fruit was measured at pull out (Year 1 n = 9; Year 2 n = 18), and the second set was placed in a dark room at 20°C for 7 d to simulate time in a supply chain and assessed texture after this ripening period (Year 1 n = 9; Year 2 n = 18). The fruit texture metrics provided by the MDT-2 penetrometer have been assessed extensively and are an accurate and reliable way to assess fruit texture [31, 32].

### 2.3 RNA extraction, quality control, and transcriptome sequencing

RNA was extracted from the ground frozen tissue using a CTAB/Chloroform protocol modified for use on pome fruit tissue in the postharvest period [33]. Extracted RNA was analyzed for purity using the NanodropOne (Thermo Fisher Scientific, Waltham, MA USA) for integrity on the Agilent Bioanalyzer (Agilent, Santa Clara, CA USA, Agilent-RNA Pico Kit, Cat #: 5067–1513), and quantity using the Invitrogen™ Qubit™3 (Thermo Fisher Scientific, Waltham, MA USA, Qubit™ RNA HS Assay Kit, Cat #: Q32852). Only RNA that met the following standards was used for downstream RNA-Seq and qPCR: A260/A280 ≈ 2.0, RNA Integrity Number (RIN) ≥ 8.0.

To generate RNA-Seq data, libraries using Lexogen’s QuantSeq 3’ mRNA-Seq Library Prep Kit FWD (Cat # 015; www.lexogen.com) were prepared at the Penn State Genomics Core Facility (University Park, PA, United States) as described in [4]. Libraries were sequenced on a 150 base pair single-end protocol to a target volume of ∼ 8–10 million reads per biological replicate on Illumina’s HiSeq 2500 in Rapid Mode. Read data are publicly available at the Sequencing Read Archive (BioProject PRJNA938164).

### 2.4 Processing of RNA-seq data for analysis

Raw RNA-seq data was preprocessed with Trimmomatic [34] to remove the leading 12 nucleotides. This trimming is recommended for QuantSeq 3’ FWD sequencing prior to genome alignment [35]. Transcripts were then aligned and counted using the GEMmaker workflow [36] using the Hisat2 [37]. The ‘Golden Delicious’ doubled-haploid genome (GDDH13) [38] was downloaded from the Genome Database for Rosaceae (GDR) [39] and used for alignment. The Hisat2 option in GEMmaker automatically runs the following bioinformatic tools: Fastqc [40], Trimmomatic [34], Hisat2 [37], Samtools [41], Stringtie [42], and MultiQC [43]. The output of this workflow is a Gene Expression Matrix (GEM) with counts reported in Transcripts per Million (TPM). The average read alignment was 71%, with an average of 53% of reads assigned unambiguously. The multiQC [43] report of the alignment is included in S2 Table in S4 File. Seven samples were removed due to low alignment (S2 Table in S4 File). A total of 46559 genes are present in the GDDH13 genome, but we did not have alignment to all of these with our dataset. Any gene which had zero RNA-seq reads was removed from this analysis. The final GEM consisted of 128 samples and 32303 genes (S1 File).

### 2.5 Random forest modeling of RNA-seq samples

The GEM and the Overall Average Hardness post-simulated supply chain (OAH) measurements were used for modeling firmness using RandomForestRegressor from the sklearn package (version 1.1.3) [44]. OAH was used as the dependent (target) variable, and the expression values of all genes in the GEM were used as the independent (explanatory) variables. The OAH is a metric of fruit flesh texture provided by the Mohr Penetrometer, and is the average flesh firmness (in pounds of pressure) across Regions 1 and 2 of pome fruit cortex [45–47]. While the industry typically uses M1 (maximum firmness in Region 1) and other metrics (including aggregated and/or proprietary ones), we use OAH because it is a convenient proxy for full fruit texture and offered more contrast in our experiment than other metrics reported by the MORH texture analyzer. It is therefore an appropriate metric to use for exploration of biomarkers that may be viable PTBs.

The RF full model (RF-fm) used all 32303 genes in the GEM and was bootstrapped 100 times. Parameters for the RandomForestRegressor were: n_estimators = 1000, max_features = 0.5, and min_samples_leaf = 5. The feature importance of each gene was recorded after each run, and the total feature importance was calculated as the sum of feature importance over 100 runs. This summed feature importance score is a measure of importance that the gene has in the prediction of firmness. Thus, the top 15 genes based on summed feature importance were selected as a reduced sample set to be used for both stability measurements and for the RF reduced model (RF-rm).

The RF-rm was created using the same parameters as the RF-fm with the exception that only the top 15 genes from the RF-fm were used. This was intended to use the most predictive genes from the previous model while reducing the chance of overfitting due to too many features. This reduced 15 gene set also represents a more realistic number of genes that could be sampled in a commercial setting.

### 2.6 Elastic net modeling of RNA-seq samples

Elastic Net (EN) feature selection was performed using the sklearn packages ElasticNetCV (version 1.1.3) [44]. As in RF, OAH post was used as the dependent variable, and TPM expression values were used as independent variables. For the full model, all 100 bootstraps were performed using randomized train test splits for each run. l1 ratios searched were 0.1, 0.5, 0.7, 0.9, 0.95, 0.99 and 1.0. Other parameters included cross-validation of 5 (cv = 5) and a maximum of 1000 iterations (max_iter = 1000). Like the RF-fm, the EN full model (EN-fm) used all 32303 genes during feature selection.

The coefficients of the best model were recorded after each iteration. The absolute value of these coefficients was taken and then normalized to a total of 1 to make coefficients comparable to RF’s feature importance. The top 15 genes were those with the highest total normalized coefficients. The top 15 genes were then used to create the EN reduced model (EN-rm). Besides the number of genes, the EN-rm was created using the same parameters as the EN-fm.

### 2.7 Stability measurements

The stability of both RF and EN models was assessed using techniques outlined in [48]. In short, genes were numerically ranked in each run according to either their feature importance (RF) or coefficients (EN). For each of the 100 runs of EN-fm and RF-fm, the top 15 genes ranks were plotted. The rank of an individual gene is expected to remain relatively the same if a model is stable and to change drastically if a model is unstable.

### 2.8 Boruta feature selection of samples

Boruta Random Forest (BRF) feature selection was performed on the mean OAH post measurements with the GEM. BRF does not attempt to make predictive models (e.g., genes predictive of firmness), but instead can be used to filter genes that may have been included due to overfitting. It uses the entire dataset to see which features perform better than a randomized “shadow feature” [49]. We use it to verify feature predictions selected from previously discussed models. BorutaPy [49, 50] was used with the following parameters: max_iter = 200, perc = 90. BRF was run 100 times on bootstrapped resampling of the data set.

### 2.9 Gene of interest selection for qPCR validation

#### 2.9.1 Criteria for gene selection

The top 45 genes identified as being predictive of texture loss from the RF regression model were selected and classified into orthogroups pre-computed with the 26Gv2.0 scaffold using PlantTribes2 [51]. Orthogroup multiple sequence alignment, phylogenetic tree estimation, homology inference, and gene model evaluation were performed using genes from 16 Rosaceae genomes (the same 15 from [52] plus *Malus baccata* [53]) plus the scaffolding species following methods from [52]. These top 45 genes were further filtered for primer development following criteria from [54], giving priority to genes in (in no particular order): 1) small orthogroups (ideally < 15 members in *Prunus persica*), 2) high expression, 3) low variance between biological replicates, and 4) those with linear expression profiles. From these criteria, a set of 15 genes were selected for primer development for a preliminary assessment of potential PTB performance in a novel sample set (Year 2). A set of 15 genes was pragmatically chosen to simulate an economically feasible ‘real world’ scenario of PTB deployment. To guide the selection of regions for targeted primer development, orthogroup multiple sequence alignments produced by PlantTribes2 were visualized and manually examined in Geneious R9 (https://www.geneious.com). Only gene regions with highly homologous sequences across apple cultivars were selected for primer design. The PlantTribes2 Orthogroup Classification and Annotations for these 15 genes in S3 Table in S4 File.

#### 2.9.2 Primer development and qPCR

Primers were developed in Geneious using the Primer3 plug-in [v2.3.4 [55]], following parameters detailed in S4 Table in S4 File. For candidate genes with highly similar homologous sequences, primer development was targeted to specific regions in alignment with the highest dissimilarity. Candidate genes and their primer characteristics can be found in S5 Table in S4 File (CDS and primer alignments in S2 File). Reference genes previously used in [56] and [4] were selected from the literature: MDP0000274900 [57], MDP0000173025 [58], and MDP0000223691 [59]. The GDDH13 homologous sequences for these reference genes were identified, using PlantTribes2, as MD09G1190100, MD16G1209000, and MD15G1211100 respectively [60]. Primers were synthesized by Integrated DNA Technologies (IDT, Coralville, IA), dissolved in qPCR-grade water (catalog no. W4502; Sigma-Aldrich, St. Louis, MO) to produce 100 μm solutions, and stored at –20°C. qPCR was performed as described in [56] for ‘Granny Smith’ on a subset of 33 Year 2 samples (corresponding to similar early and late postharvest storage time points from each experimental treatment and condition in Year 1—S6 Table in S4 File) with a slight modification to the protocol: the qPCR reaction volume was increased to 15 μL [to accommodate automated liquid handling by an epMotion 5073 (Eppendorf, Hamburg, Germany)] by increasing the volume of SYBR per reaction while maintaining the template mass per reaction (10 pg cDNA).

#### 2.9.3 qPCR post-processing for normalized expression

Amplification and melt curves for each triplicated group of technical replicates were manually inspected for Ct variance and melt curve anomalies. Individual technical replicates were removed from downstream analyses if Ct variance was > 1.5. Next, reaction efficiency was calculated based on raw amplification data using the R v4.1.2 [61] package ‘qpcR’ v1.4–1 [62]. First, statistical model selection was performed for sigmoidal fit testing of the raw real-time PCR data for the reference gene PCR runs using the *mselect*() function with comparing nonlinear sigmoidal models (l4, l5, b4, b5) and exponential models (expGrowth, expSDM, linexp), a bilinear model (lin2), and a mechanistic model (cm3) [63]. The *mselect*() function had the following parameters set: fctList = list(l5, l4, b5, b4, cm3, lin2, linexp, expGrowth, expSDM), crit =“weights”. The best overall models (lin2 and linexp) were selected based on model goodness of fit Akaike Information Criterion (AIC) and r^2^. Using the best model, the *modlist*() function was rerun, with the following parameters set: remove =“none”, smooth = “spline”. Threshold cycles were determined using the ‘Cy0’ method [64] to calculate efficiency using the *pcrbatch*() function. In the *pcrbatch*() function output, the efficiency was calculated using the best overall model (lin2 or linexp) on a gene-by-gene basis. Computed reaction efficiencies were entered into their corresponding PTBs in BioRad’s CFX Maestro 1.0 software (4.0.2325.0418) and used to calculate relative normalized expression values with the Pfaffl method [65] and three reference genes. Reference gene stability was assessed with the CFX Maestro software using the ‘Reference Gene Selection Tool’ (CFX Maestro™ Software User Guide v1.1), as well as a manual, visual assessment of expression across samples. One reference gene, MD15G1211100, was observed to have moderate variation across the samples, and was flagged by the ‘Reference Gene Selection Tool’ as having ‘Acceptable’ Stability (as opposed to ‘Ideal’ stability. This reference gene was removed from analysis prior to normalized expression computation.

### 2.10 Literature genes random forest

Genes were selected from previous literature concentrating on loss of firmness and texture in apple fruits [66–69]. If necessary, literature gene names were converted from other apple genomes nomenclature (i.e. [70]) to GDDH13 [38] using homology through OrthoFinder [71]. In total, 98 genes from the literature were identified (S7 Table in S4 File). 85 of these genes had at least one read aligned to them in the GEM, and were used to create RF models in the same manner as previously discussed. 100 bootstrap replicates were performed using the 85 gene set, and the total feature importance of each gene set was determined. The genes with the top 15 summed feature importance were then used in a new model, with model performance and feature importance being calculated.

## 3 Results and discussion

### 3.1 Firmness loss

**Fig 2:**
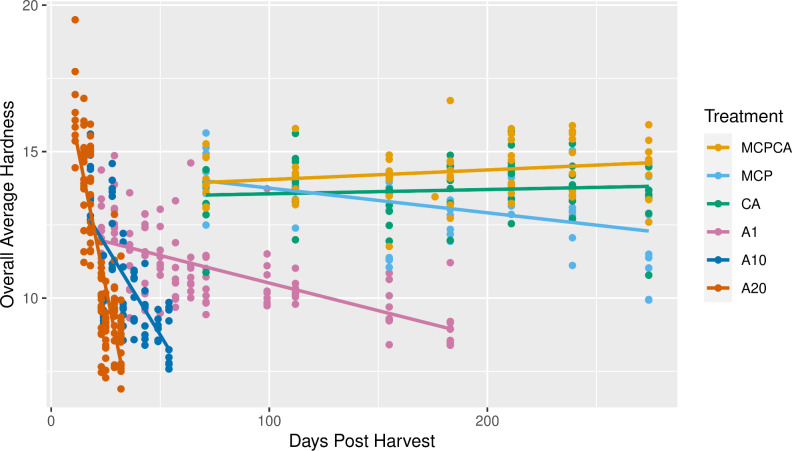
Overall average hardness after a 7d simulated supply chain (in air at 20°C).

Firmness declined more rapidly in the short-term fruit than in the long-term fruit. Fruit stored at room temperature of 20°C (A20) lost firmness most quickly, followed by fruit stored at 10°C (A10) and 1°C fruit (A1). Long-term fruit maintained firmness throughout the experiment (Fig 2). These firmness trends are consistent with previous knowledge of apple ripening [72].

### 3.2 Transcriptomic data pre-processing

**Fig 3:**
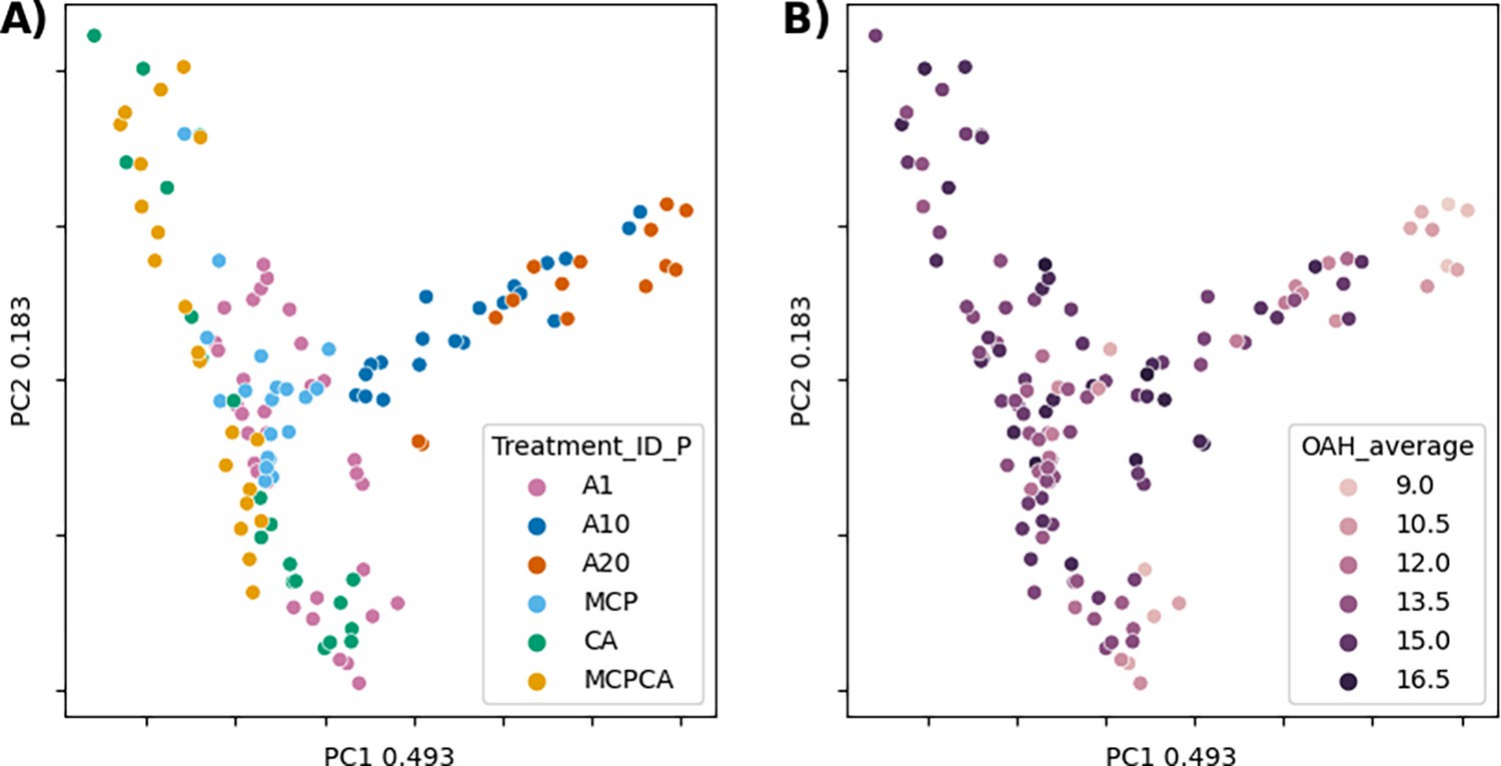
PCA 1 and 2 of TPM transcriptomic data counts colored by A) treatment and B) days post harvest. PC1 primarily separates fruit based on temperature and days post-harvest of the warmer fruit whereas PC2 separates based on days post harvest of fruit treated with 1-MCP and CA.

A principal component analysis (PCA) of the TPM (transcript per million) read counts shows that the expression data is separated primarily based on storage temperature (1°C, 10°C, 20°C) along PC1, and by storage condition (1-MCP and CA) along PC2 (Fig 3A). This shows that we have variance within the transcriptome that describes both experimental conditions and days after harvest. Coloring with Days Postharvest also shows considerable variation over the PCA plots (Fig 3B).

### 3.3 Model performance—random forest vs. elastic net

**Fig 4:**
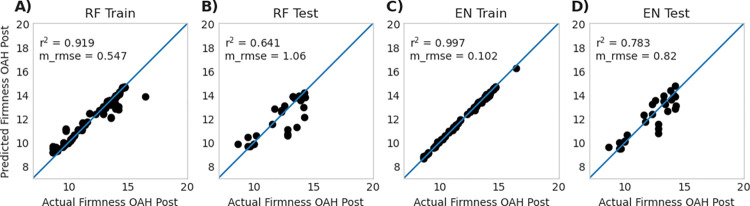
Model performance for a single run of Random Forest full model (RF-fm) **(A** and **B)** and Elastic Net full model (EN-fm) **(C** and **D)** using all genes. The data used in each model is split between train (RF-fm **A** and EN-fm **C**) and test data (RF-fm **B** and EN-fm **D**). Data for replicated 100 runs of these models is presented in Table 1. Reported r^2^ and m_rmse values in this figure represent a single run of a representative model, whereas data reported in Table 1 represents the average of 100 replicates.

The first step was to create models using the full feature set of genes which we refer to as full models. Model performance for the Random Forest full model (RF-fm) and the Elastic Net full model (EN-fm) were comparable (Table 1), with testing r^2^ for EN-fm (r^2^ = 0.767 ± 0.099 SD) performing better than RF-fm (r^2^ 0.687 ± 0.124 SD). A visualization of a single run of both EN-fm and RF-fm is visualized in Fig 4 which is split into training (Fig 4**A** for RF-fm and Fig 4**C** for EN-fm) and testing (Fig 4**B** for RF-fm and Fig 4**D** for EN-fm) sets.

**Table 1:** [*2-column fit table]* r^2^ and m_rmse values with standard deviation of 100 bootstrap runs for all models. For Random Forest and Elastic Net ‘All Genes’ summary, the ‘Full Models’ use all genes from data (32303) whereas the ‘Reduced Models’ use only the top 15 genes from the full model. The Random Forest Literature full model was done with 85 genes referenced in S7 Table in S4 File, and the reduced model was run with only the top 15 genes from this subset. The models run for qPCR used only the 15 genes selected for qPCR, therefore a reduced model was not applicable. Performance metrics are reported with ± standard deviation.

**Random Forest All Genes**				
	r^2^ Train	m_rmse Train	r^2^ Test	m_rmse Test
Full Model	0.928 ± 0.007	0.506 ± 0.025	0.687 ± 0.124	1.02 ± 0.216
Reduced Model	0.887 ± 0.012	0.633 ± 0.039	0.727 ± 0.099	0.954 ± 0.19
**Random Forest Literature Genes Only**				
	r^2^ Train	m_rmse Train	r^2^ Test	m_rmse Test
Full Model (85 Genes)	0.897 ± 0.008	0.606 ± 0.027	0.68 ± 0.134	1.02 ± 0.215
Reduced Model	0.882 ± 0.009	0.647 ± 0.026	0.711 ± 0.106	0.992 ± 0.209
**Random Forest qPCR Genes Only**				
	r^2^ Train	m_rmse Train	r^2^ Test	m_rmse Test
15 Gene Model	0.897 ± 0.011	0.604 ± 0.03	0.748 ± 0.077	0.925 ± 0.15
**Elastic Net All Genes**				
	r^2^ Train	m_rmse Train	r^2^ Test	m_rmse Test
Full Model	0.949 ± 0.057	0.311 ± 0.295	0.767 ± 0.099	0.889 ± 0.184
Reduced Model	0.85 ± 0.011	0.731 ± 0.025	0.784 ± 0.061	0.845 ± 0.098

In addition to the RF-fm and EN-fm, Boruta Random Forest (BRF) was also performed. BRF selects “all relevant” genes using randomized shadow features. If a gene does not perform better than a shadow feature for predictions, it is eliminated [49]. Importantly, BRF does not attempt to predict firmness but instead concentrates on identifying all relevant features which are able to predict firmness better than a shadow feature. Out of 100 bootstrap runs of BRF on randomized train test sets, 51 genes were selected as performing better than shadow features (S8 Table in S4 File). Twelve of the top 15 genes from the RF model (based on feature importance rank) were present in the BRF 51 genes set. This overlap indicates that, for this data, the feature selection for RF prediction models is consistent with BRF feature selection models [73]. A strong overlap indicates that the features selected by RF are sufficient for predicting actual firmness in this dataset rather than just fitting on noise. None of the top 15 genes from EN (based on normalized coefficients) were represented within the BRF gene set.

### 3.4 Model stability

**Fig 5:**
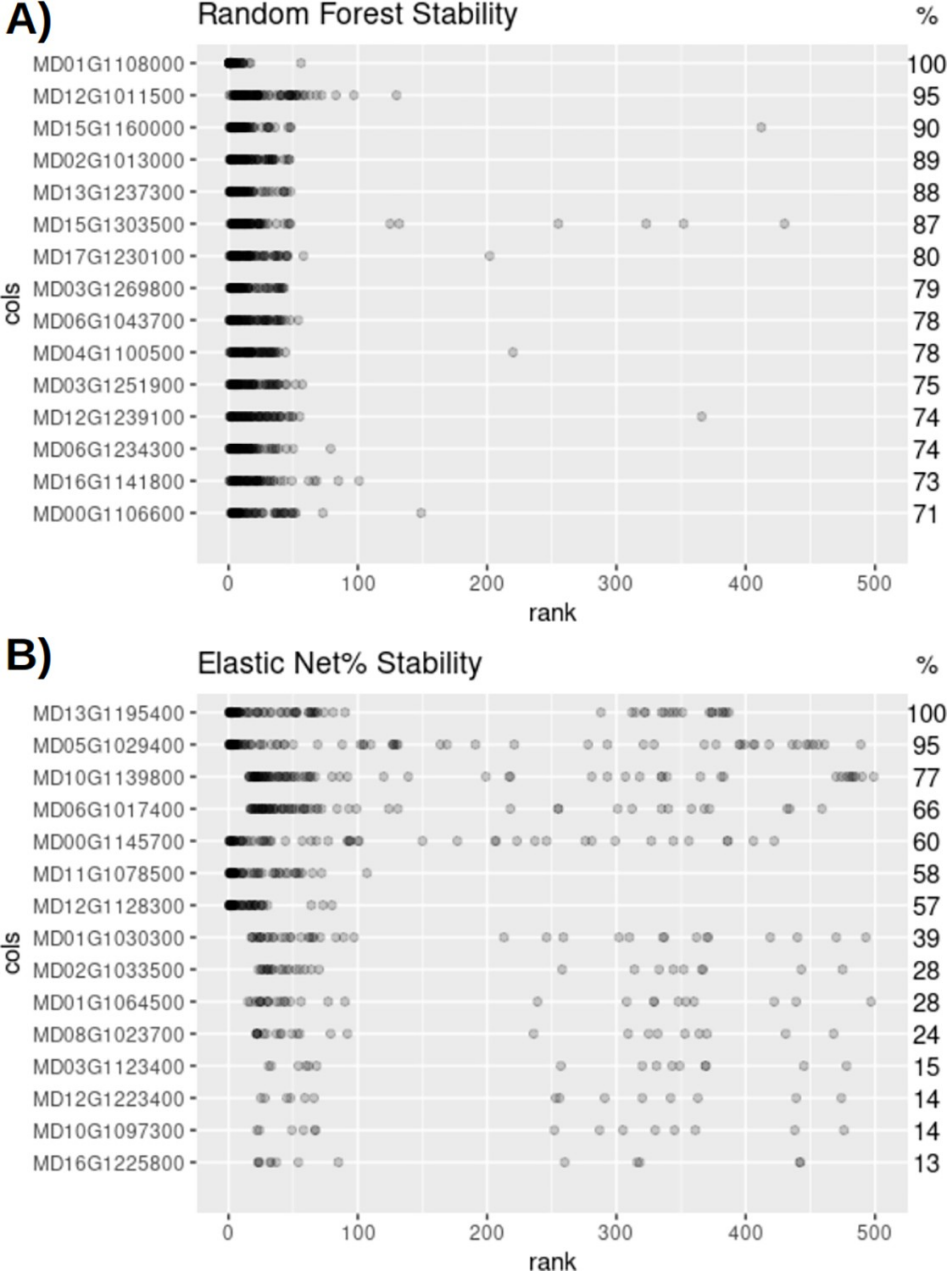
Model stability of A) Random Forest (RF) Full Model (FM) and B) Elastic Net (EN) FM. The top 15 genes of each model are shown along the y-axis. Each point represents a single bootstrap re-run of the model and its position along the x-axis is the gene’s rank in importance from the re-run model. A rank of 1 is given to a gene if it is the most important in the respective model. A point is present for a re-run model only if the gene occurred in the top 500 important genes. Numbers on the right side y-axis of the graph indicate how many times the gene was selected by the model.

While RF-fm and EN-fm performed similarly in terms of r^2^ and m_rmse, stability differed. The stability of each model was determined by bootstrap re-sampling the data and re-running the model 100 times [48]. After each run, features were ranked by importance, and the variance of the ranks of the top 15 RF-fm and EN-fm genes was explored. The RF-fm was more stable than the EN-fm when compared across 100 runs. The feature importance ranks these runs are visualized in Fig 5A for the top 15 genes of the RF-fm and Fig 5B for the EN-fm. Each point in the figure represents the rank of the gene from a single bootstrap run. Genes with high importance for, or that are more predictive of firmness will have a lower rank and appear as a point on the left-hand side of the plot and the variance of rank can be visualized by the spread of points along the x-axis. The left-hand y-axis lists the top 15 genes in the model. In some of the bootstrap runs, the top 15 genes were not ranked within the top 500 genes. The frequency that each gene appeared in the top 500 is indicated on the right-hand y-axis. RF-fm genes on average appeared in 82.07 (+/- 8.83 SD) out of 100 bootstrap runs, whereas EN-fm genes appeared in 45.87 out of 100 (std 29.78) which was significantly less than the RF-fm (Unpaired t-test p < 0.0001). The minimum number of times one of the top 15 genes was selected in the RF-fm was 71, compared to 13 in the EN-fm. Fig 5 also shows that top genes in the EN-fm had a larger spread of rank when compared to RF-fm which displayed lower variability for all genes. Neither method selected all top genes in respective models every time.

### 3.5 Model performance of top genes

The top 15 genes from each model were used to create new models, referred to as reduced models, to explore if a model using a small subset of genes could perform as well as the full model (32303 genes). This was done for two reasons, first, to simulate a realistic number of genes that could be sampled economically in a commercial setting, and second to help improve model performance. A smaller feature set can cause a model to be more generalizable and reduce the chance of overfitting [74]. Generalizability is desirable as this can make models more robust to inherent variability when applied in novel testing data due to, for example, environmental variation among years and orchards.

**Fig 6:**
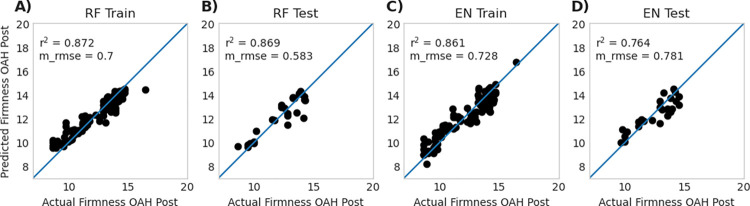
Model performance of a single random forest reduced model (RF-rm) and elastic net reduced model (EN-rm) training **A** and testing **B.** training **C** and testing **D**. Data for replicated 100 runs of these model is presented in Table 1. Reported r^2^ and m_rmse values in this figure represent a single run of a representative model, whereas data reported in Table 1 represents the average of 100 replicates.

Both EN-rm and RF-rm had increased performance within the testing data when compared with EN-fm and RF-fm (Table 1), with RF-rm achieving r^2^ of 0.727 ± 0.099 in the testing set and EN-rm achieving r^2^ of 0.784 ± 0.061. The reduced number of genes allowed the models to be more generalizable than full models and illustrates the importance of feature reduction when dealing with datasets that have many features. The performance of a single run that is representative of most runs of both RF-rm and EN-rm is visualized in Fig 6.

Both reduced models performed similarly, but there were differences in the genes selected for each. The genes which were selected for RF-rm and EN-rm do not overlap with each other and exhibit different expression patterns. Several of the genes selected by EN have low expression levels. The TPM expression levels of these top 15 genes are visualized in S2 Fig for RF and S3 Fig for EN.

### 3.6 Literature genes random forest

**Fig 7:**
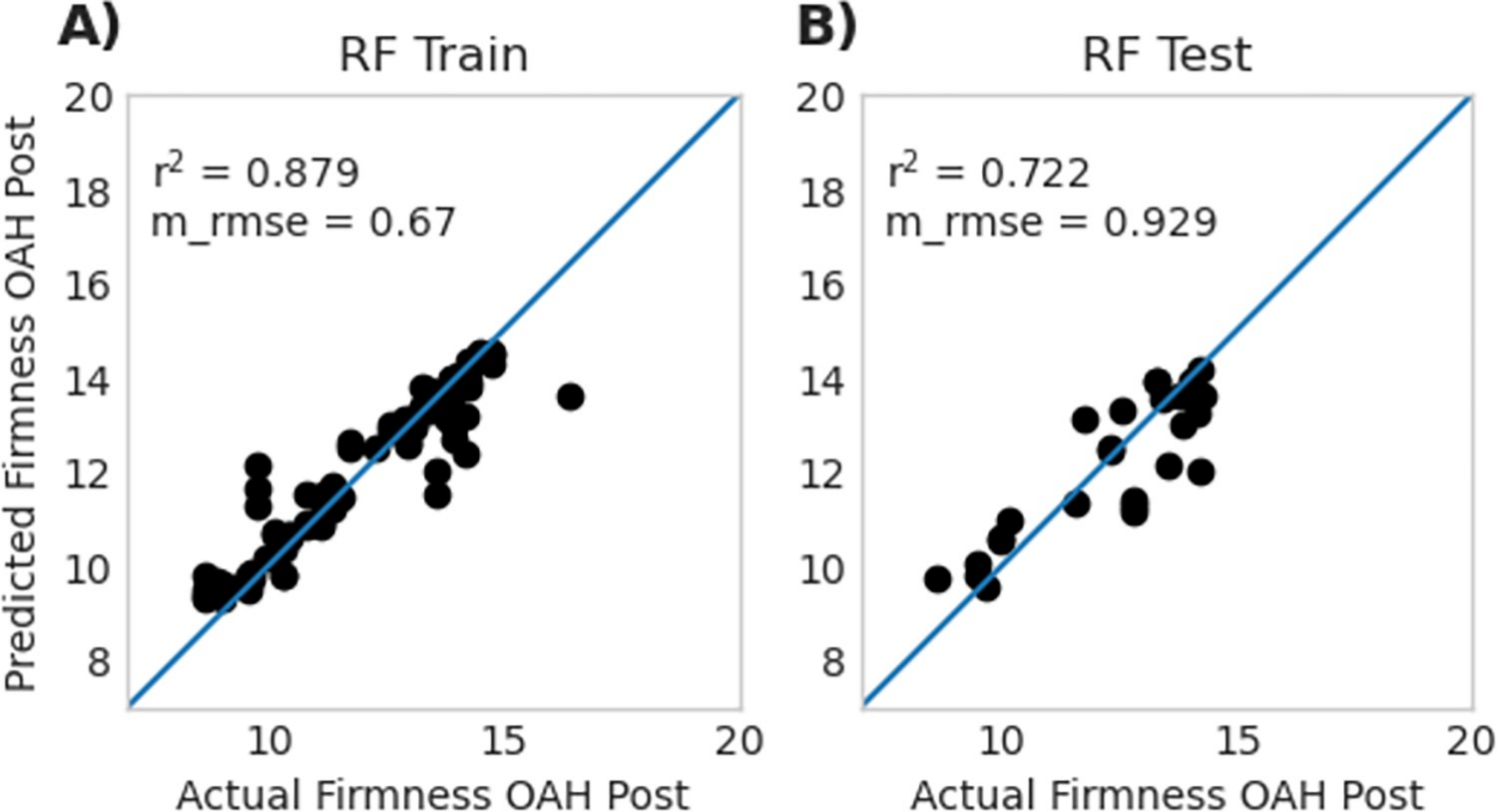
[*1-column fit image]* Random Forest model for the top 15 literature genes selected from the literature gene full model (85 genes). **A** is training data and **B** is testing data. The literature models had reduced performance compared to models using all genes. Data for replicated 100 runs of this model is presented in Table 1. Reported r^2^ and m_rmse values in this figure represent a single run of a representative model, whereas data reported in Table 1 represents the average of 100 replicates.

Random Forest model performance was also assessed for a set of 85 genes related to firmness and other texture traits identified from the literature. There were no genes identified from the literature that also appeared in the top 15 genes identified by any of the previous models. Literature genes were assessed using two models. The full model used the 85 genes referenced in S7 Table in S4 File and the reduced model used the top 15 genes from the initial 85-literature gene model. Both models were comparable, with statistics on r^2^ and m_rmse present in Table 1. The performance of a single run of the literature-reduced model is visualized in Fig 7. Both models had slightly lower performance than models created using the entire dataset (Table 1). Gene expression patterns and gene names from literature for the top 15 literature genes used in the reduced model can be seen in S4 Fig.

We suspect that the slightly lower performance of the models using genes from the literature may be due to some of the genes being transcription factors that are only turned on for short periods of time and that this gene set is expressed prior to firmness loss [66–69]. However, the fact that these genes were able to perform comparably to our models indicates the depth of information contained within RNA-seq datasets and the power of RF models.

### 3.7 Exploration of qPCR for model evaluation

#### 3.7.1 qPCR validation data

A commercially viable PTB must consist of a tractable number of targets, in this case mRNA transcripts, for the test to be feasible. Here, we performed a targeted evaluation using qPCR of 15 genes (selected from the RF-fm) in a fully independent replication of the initial experiment (i.e., Year 2 samples). A positive, significant correlation between RNA-seq and qPCR measurements would indicate that predictive genes identified by our models are more likely to be robust across different sample sets (i.e., years, orchards, data types) and could be investigated further for future PTB development. Eight of the 15 selected genes had Pearson correlation coefficients over 0.67, with three of these over 0.90. However, the other seven genes evaluated showed low, non-significant correlations (p > 0.05). These trends comparing the qPCR data from 2019 and the transcriptome data from 2018 are visualized in S5 Fig. The performance of some of these preliminary PTBs indicates that predictability scores in our models can translate to ‘real world’ predictability using qPCR technology—a promising first step. However, almost half of the tested PTBs performed poorly. We may be able to improve the number of high performing genes by selecting other genes with high feature importance that maintain high predictability in the model and which can be substituted for these poorly performing genes.

To explore why some genes may have a higher agreement than others, we considered expression patterns between RNA-Seq and qPCR on a treatment-by-treatment basis (S3 File). Generally, when considering all PTBs and the overall expression patterns, the concordance is low, as indicated by global correlation analyses above. However, as we focus on specific treatment comparisons, there are apparent patterns. Fruit stored in air and not treated with 1-MCP or stored in CA had generally had higher agreement between RNA-Seq and qPCR measurements, with the A10 treatment having the most similar expression patterns overall. When CA and 1-MCP treatments were considered, the agreement between expression patterns of these two sample sets was more tenuous, with the MCPCA treatment generally having the lowest concordance. Breaking expression pattern comparisons down by time revealed indications that the length of storage and the timing of expression assessment may influence the predictive ability of the assessed biomarkers (S3 File). Notably, fruit stored in air and assessed early in the storage period (Harvest to Early Postharvest) had higher agreement between sample sets than fruit stored in CA and treated with 1-MCP. When comparisons of longer time intervals were considered (Early to Late Postharvest or Harvest to Late Postharvest), the agreement between technologies improved, especially for fruit stored in CA and/or treated with 1-MCP.

Taken all together, observed discrepancies between the expected (model) and observed (qPCR) performance of preliminary PTBs assessed here indicate that more testing is required and there are additional variables that need to be considered and incorporated into the models for enhanced performance. PBT efficacy may be influenced by a variety of factors such as maturity at harvest, annual weather patterns, other physiological indices, etc., that warrant further investigation. Furthermore, it is possible that certain GOIs identified in the models are better suited for certain conditions or applications than others, given the dynamic nature of gene expression in pome fruits during postharvest storage in modified storage conditions [56, 75–77]. Thus, the development of a set of commercially viable PTBs with predictive value is likely to be possible but requires further exploration and validation.

#### 3.7.2 Model evaluation of genes selected for qPCR

**Fig 8:**
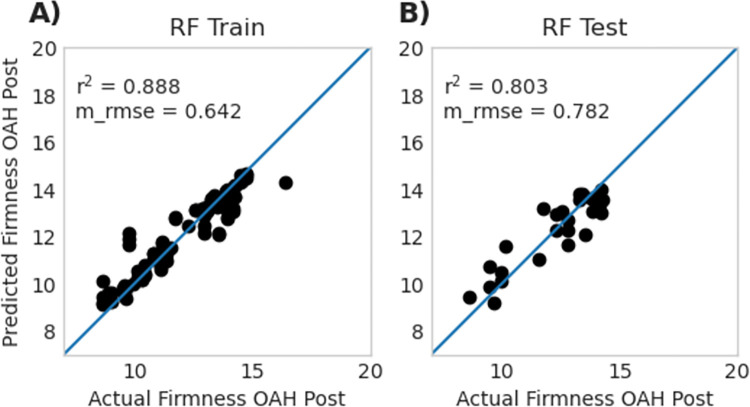
Single RF Model performance of genes selected for qPCR using the RNA-seq 2018 dataset. Data for replicated 100 runs of this model is presented in Table 1. Reported r^2^ and m_rmse values in this figure represent a single run of a representative model, whereas data reported in Table 1 represents the average of 100 replicates.

For qPCR, we selected 15 genes out of the top 30 genes from the RF-fm, about half of which were within the top 15 most important genes. To assess if model performance would drop further if ‘less’ important genes were included, we ran the Random Forest model using the 15 genes selected for qPCR (Random Forest qPCR Genes from Table 1). The performance for this gene set was 0.897 ± 0.011 SD for the training data and 0.748 ± 0.077 for the testing data, which was like the performance of the top 15 overall gene model (RF-rm, Table 1). The performance of a single run of the qPCR model is visualized in Fig 8. This provides another layer of evidence to suggest that the model is not overfitting and that despite variation in years, correlation holds. Second, because the set of genes used for qPCR had a wider range of importance scores (selected from the top 30 genes from the RF-fm) and these genes still performed well in the RF model, it implies that there may not be a single set of genes that are predictive of the phenotype being investigated. This is important for future PTB development because it allows for flexibility in terms of gene selection; other pragmatic criteria could be considered, such as signal-to-noise ratios, variability, and ease of testing.

## 4 Conclusions

Our results provide answers to several key questions about PTBs. First, these results provide more evidence supporting that gene expression profiles can be used in models that predict outcome. We identified a putative set of prognostic transcriptomic biomarkers (PTBs) capable of predicting postharvest fruit texture in ‘Gala’ apples within our experiment that included a range of commercially relevant postharvest treatments. Second, we explored two popular association methods and show that PTBs identified from a Random Forest regression-based feature selection model outperformed Elastic Net Regression. Importantly, feature set stability varied across different train-test splits indicating a propensity for error in the Elastic Net models. This work shows that Random Forest regression in ‘Gala’ apples outperformed putative firmness genes identified in the literature as associated with fruit texture. This illustrates the value of using an *ab initio* approach for PTB rather than relying solely on current molecular knowledge, which may be incomplete in non-model organisms such as apple. Third, we show that, in our data set for ‘Gala’ apple, as few as 15 genes could be used to predict firmness, with similar performance to the full predictive models that include 100’s of genes. We demonstrate the feasibility of using qPCR markers as a direct application of putative PTBs, although we stress that much more testing and development is required before a deployable set of PTBs could be delivered for ‘Gala’, let alone other apple cultivars.

While our results show that transcriptomics can be used to identify putative predictive biomarkers of traits that are highly impacted by the environment, it should be noted that this study is not exhaustive. Fruit texture in apples is well understood and controllable using common postharvest practices and the models developed in this study should be regarded as a proof of concept and a promising first step towards identification of PTBs in complex fruit quality traits (i.e. fruit texture) for ‘Gala’ apple. Importantly, this paper provides answers to questions that will be useful for anyone seeking to develop biomarkers using gene expression. More research is needed to understand other limitations (i.e., sample size requirements), and how other factors (such as gene expression normalization) affect model robustness. Because the number of environmental variables experienced by apple fruit throughout the production environment is large, more data will be needed before PTBs for complex fruit quality traits can be used in an applied setting. Further work in this area may yield information that can be used to expand the postharvest toolkit for managing apple fruit quality, leading to increased supply chain efficiency and less waste. Moreover, because gene expression is ubiquitous across all living organisms, PTBs show promise as a tool for any species with traits that are highly impacted by the environment.

## Supporting information

S1 File Compressed RNA-seq gene expression matrices. Gene expression matrices of *Malus domestica* fruit reported in this study. Counts are reported as Transcripts per Million (TPM) reads.
(ZIP)

S2 File Compressed GOI primer alignment results. Alignment files of the 15 primers designed for qPCR verification in this study.
(ZIP)

S3 File qPCR vs RNA-seq pattern comparison results. Compressed series of plots showing the difference between qPCR and RNA-seq patterns of the selected 15 genes.
(ZIP)

S4 File Supplemental tables S1-S8. (XLSX)

S1 Fig Harvest boxplots. A) Differences in fruit diameter at harvest between 2018 and 2019. B) Differences in creep at harvest during the same period.
(PNG)

S2 Fig Random forest top15 gene expression. The expression level (y-axis) of genes using TPM normalized counts over time (x-axis) for the top 15 genes identified using the random forest model.
(PNG)

S3 Fig Elastic net top 15 gene expression. The expression level (y-axis) of genes using TPM normalized counts over time (x-axis) for the top 15 genes identified using the elastic net model.
(PNG)

S4 Fig Literature genes expression. The expression level (y-axis) of genes using TPM normalized counts over time (x-axis) for the top 15 genes identified from the literature.
(PNG)

S5 Fig RNA-Seq TPM vs qPCR. Scatterplot of gene expression (y-axis) using TPM normalized counts vs qPCR levels (x-axis).
(PNG)
