# Correction: Towards identification of postharvest fruit quality transcriptomic markers in *Malus domestica*

**DOI:** 10.1371/journal.pone.0306187

**Published:** 2024-06-21

**Authors:** John A. Hadish, Heidi L. Hargarten, Huiting Zhang, James P. Mattheis, Loren A. Honaas, Stephen P. Ficklin

There is an error in the original article regarding the corresponding authorship. The last two authors, Loren A. Honaas and Stephen P. Ficklin, should both be noted as corresponding authors for this article. Loren A. Honaas’s email address is loren.honaas@usda.gov. The ORCID iD for Loren A. Honaas is 0000-0001-9187-8254 (https://orcid.org/0000-0001-9187-8254).

## References

[1] Hadish JA, Hargarten HL, Zhang H, Mattheis JP, Honaas LA, Ficklin SP (2024) Towards identification of postharvest fruit quality transcriptomic markers in *Malus domestica*. PLoS ONE 19(3): e0297015. doi: 10.1371/journal.pone.0297015 38446822 PMC10917293

