# Supplementary figures and images for: Towards identification of postharvest fruit quality transcriptomic markers in Malus domestica
Source: PLoS One. 2024 Mar 6;19(3):e0297015. doi: 10.1371/journal.pone.0297015 (PMC10917293; doi:10.1371/journal.pone.0297015)

## Slide 1
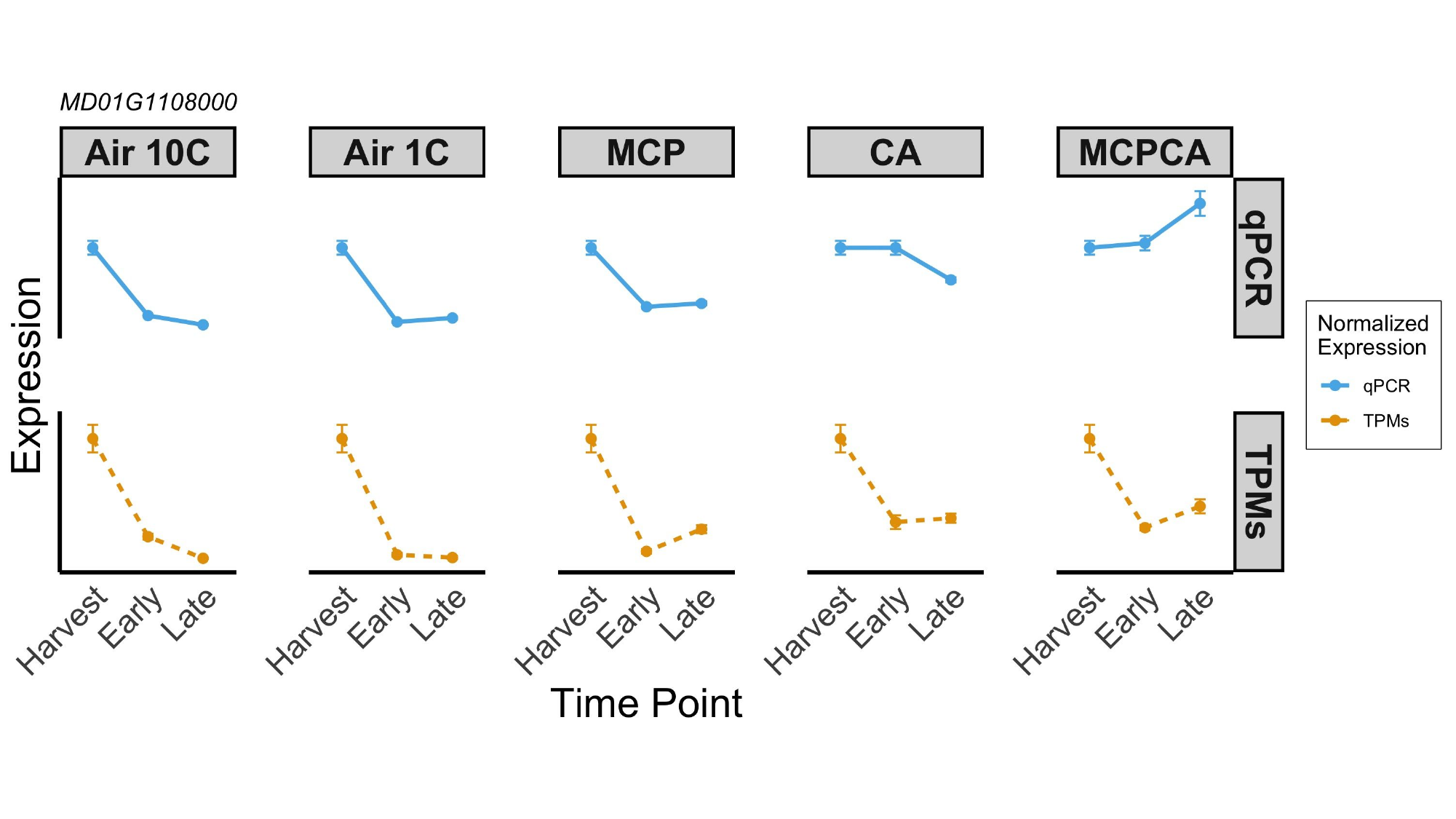

## Slide 2
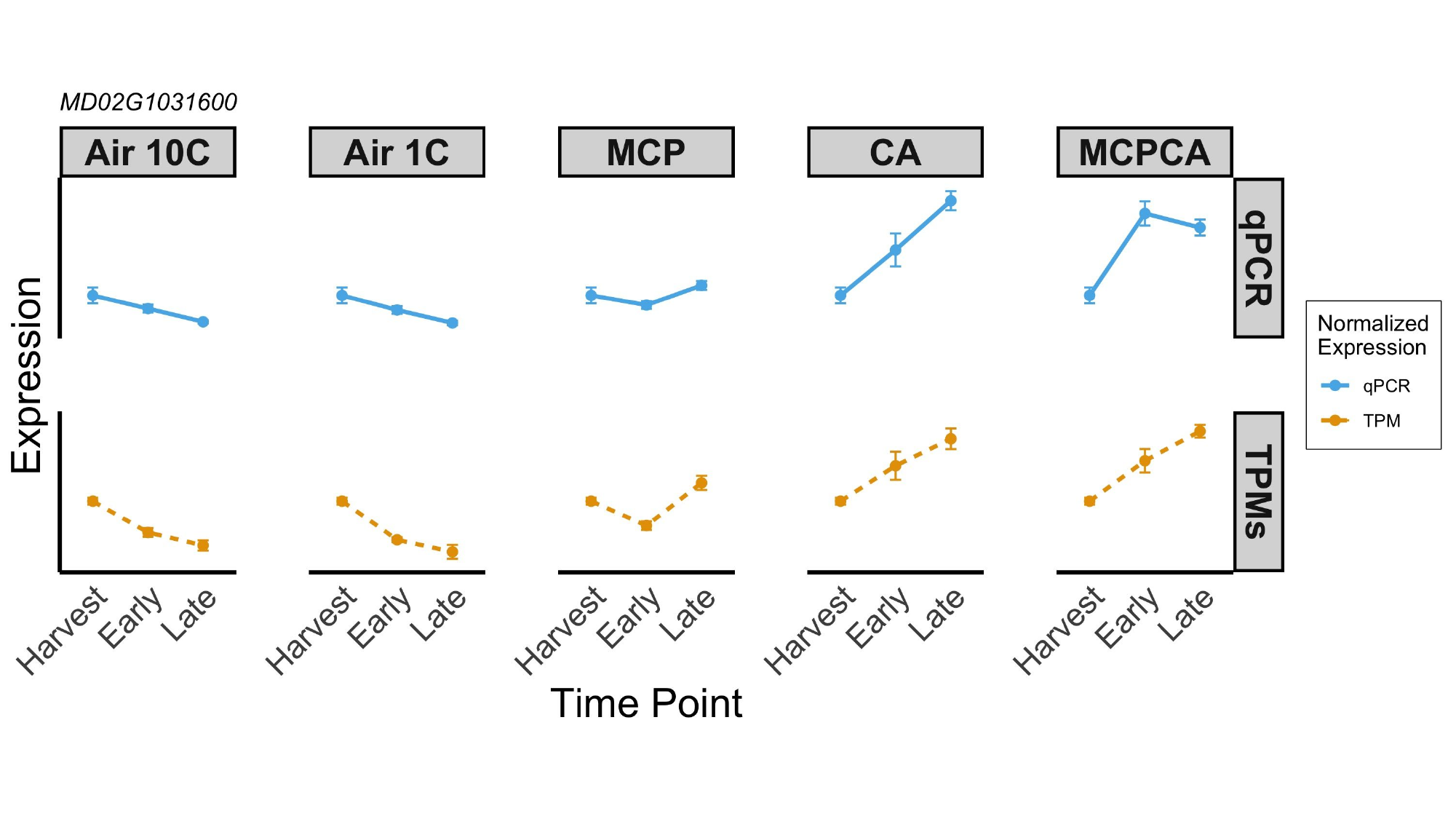

## Slide 3
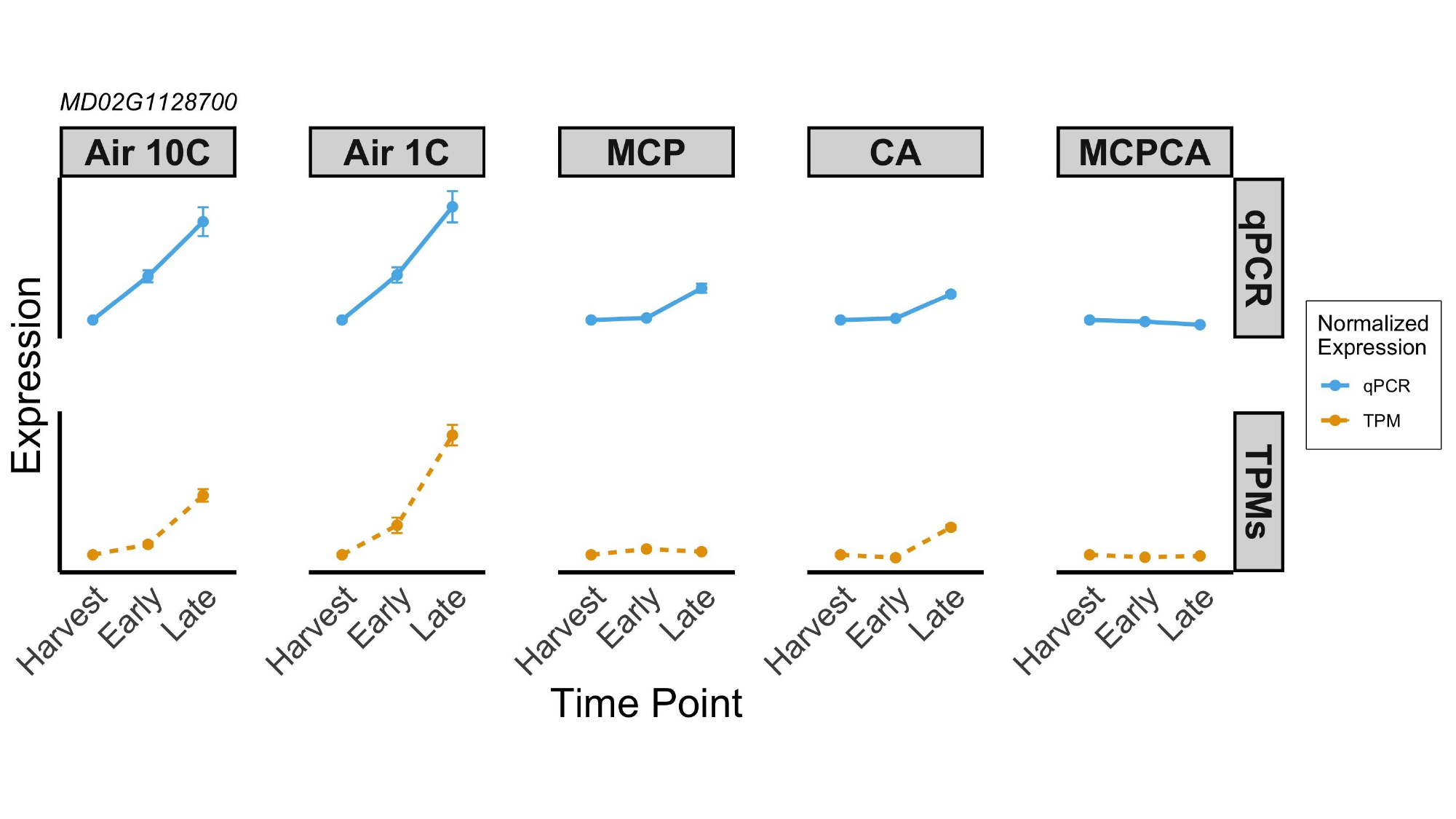

## Slide 4
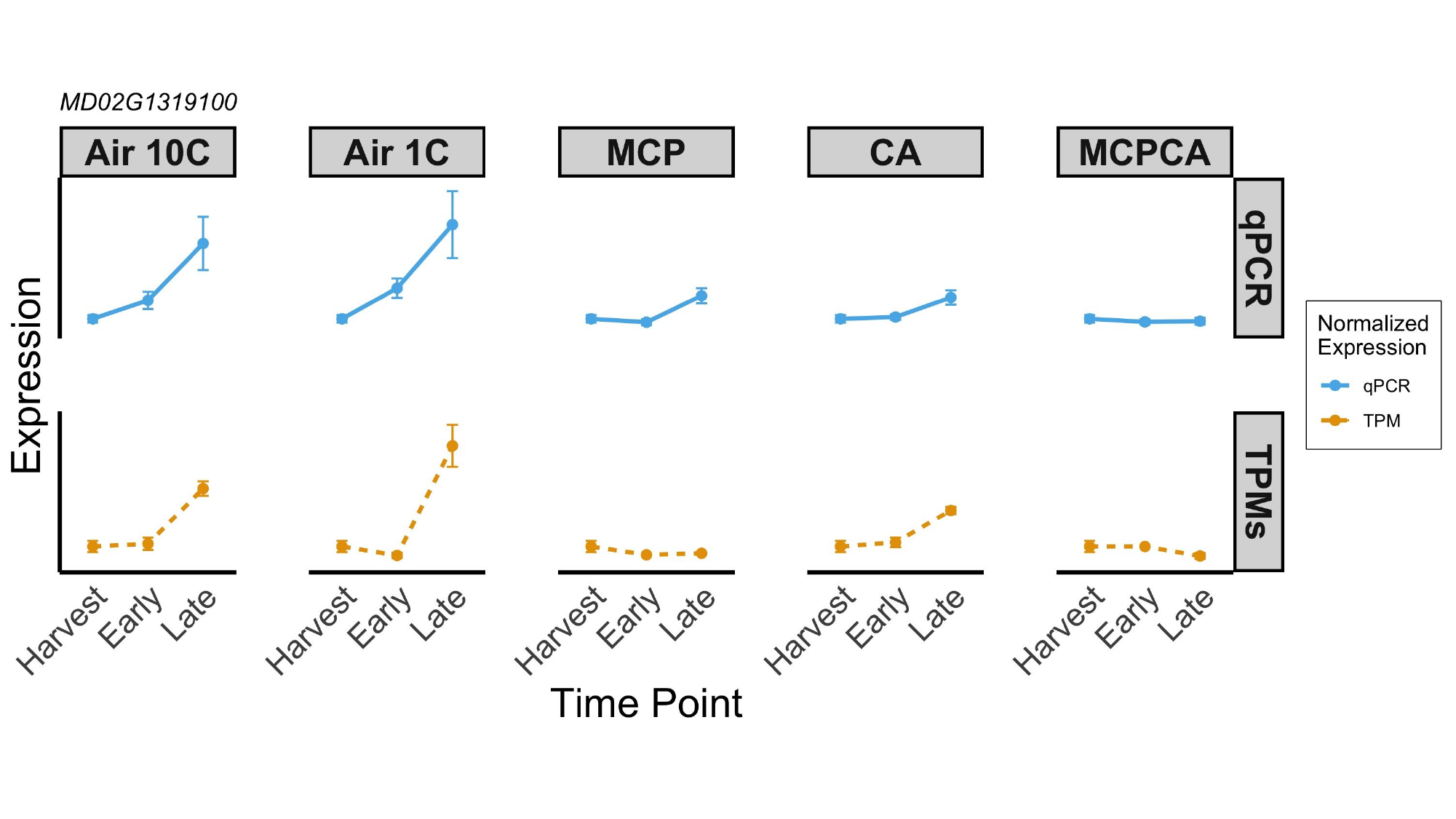

## Slide 5
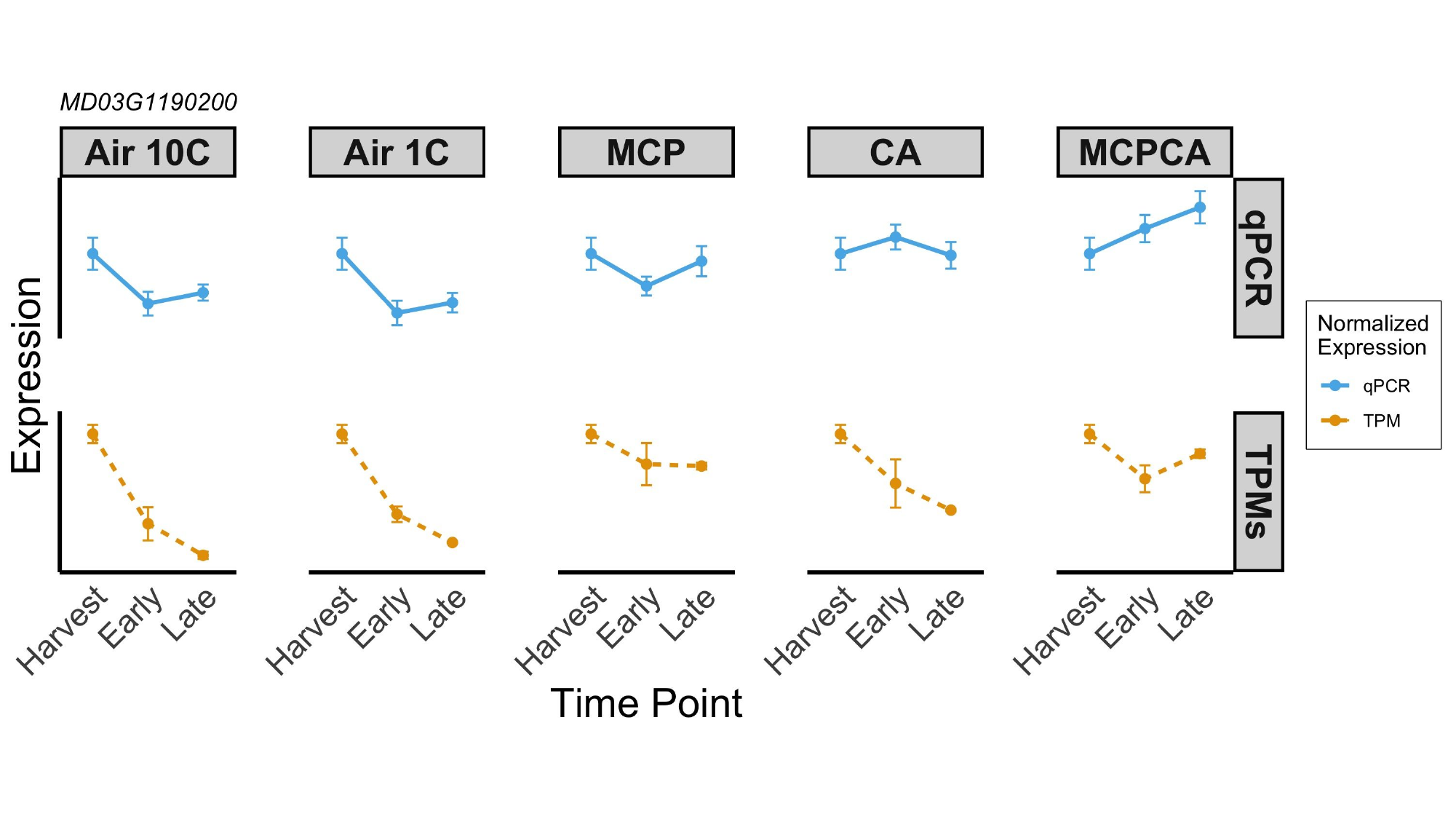

## Slide 6
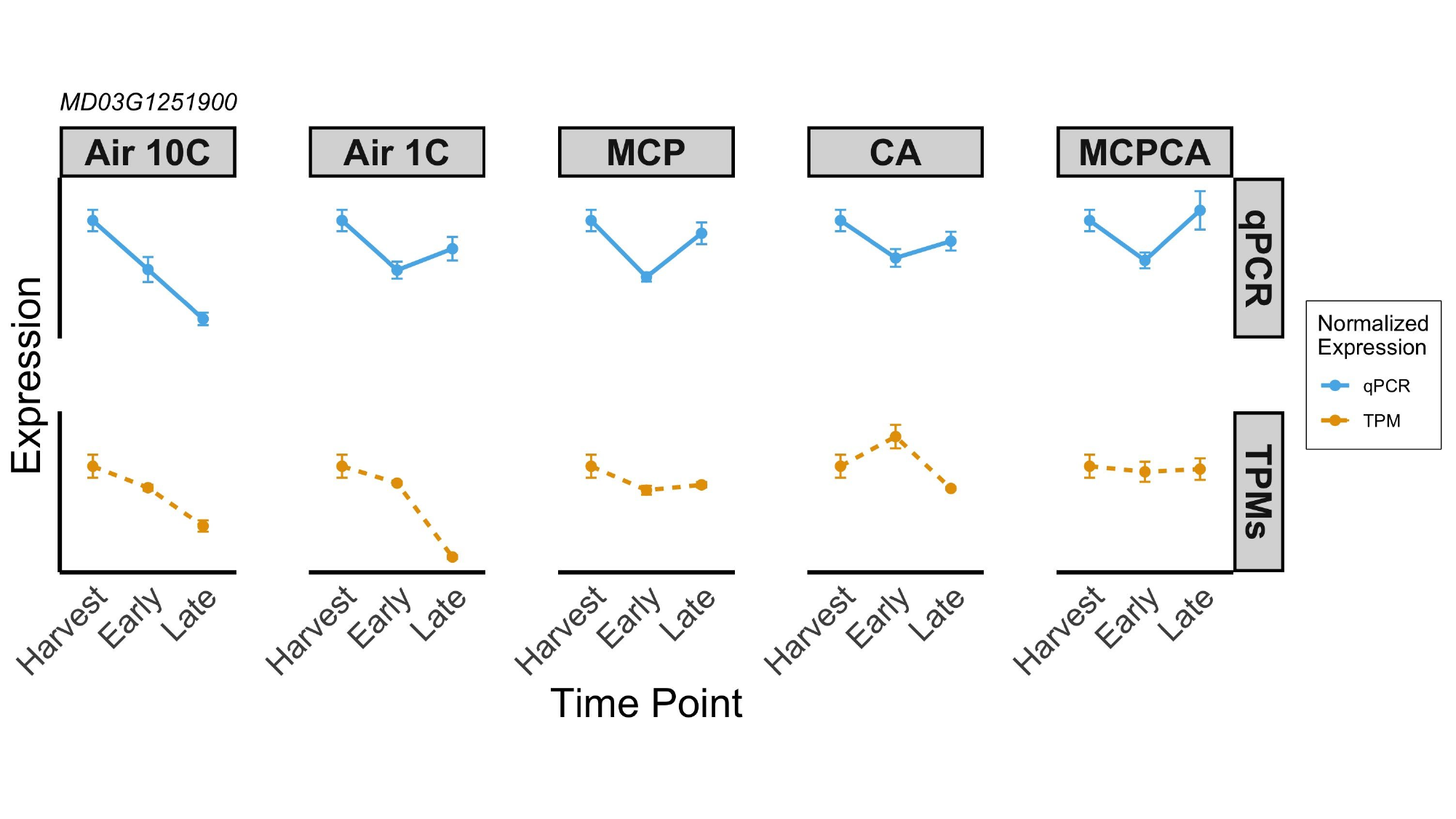

## Slide 7
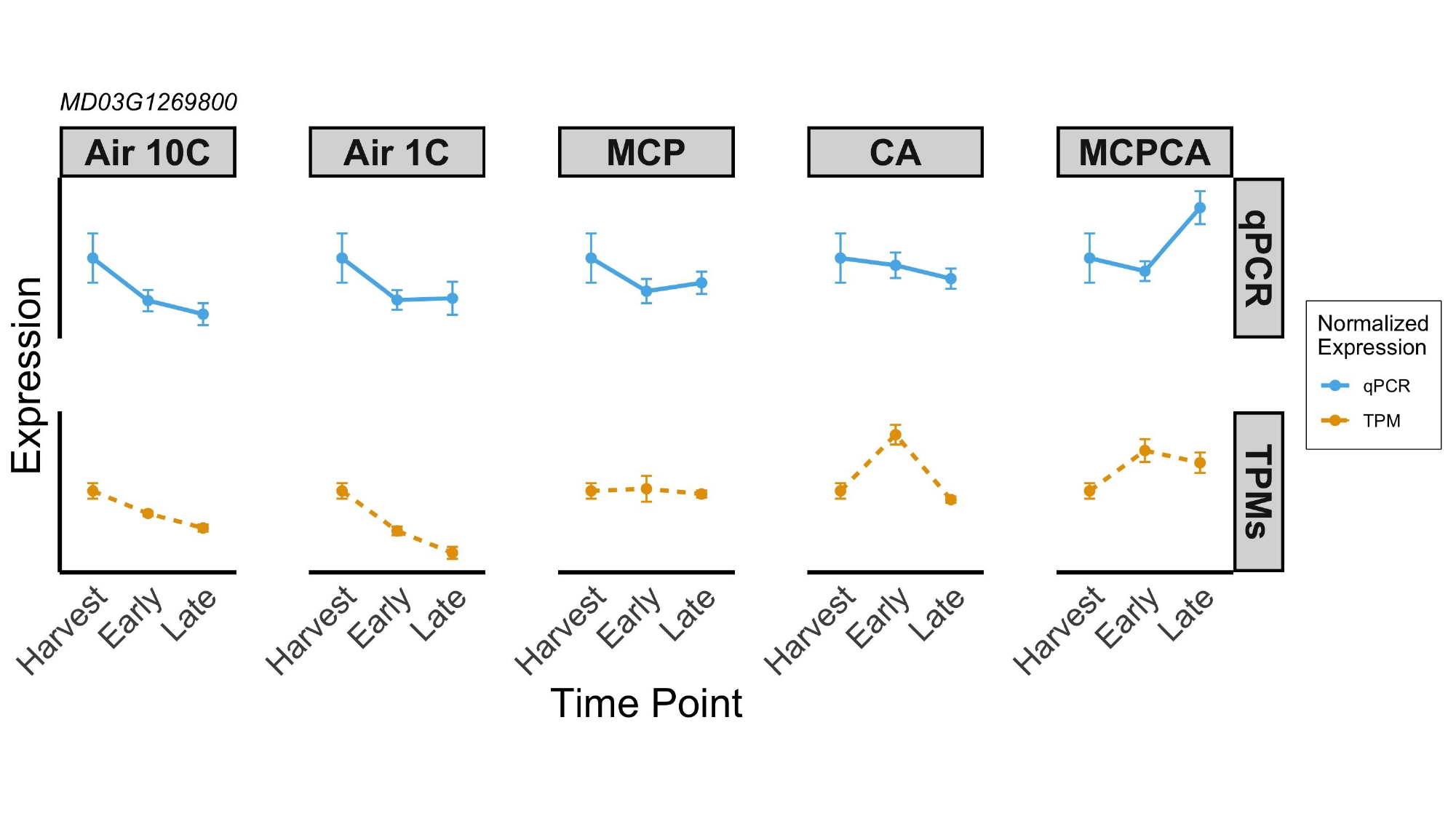

## Slide 8
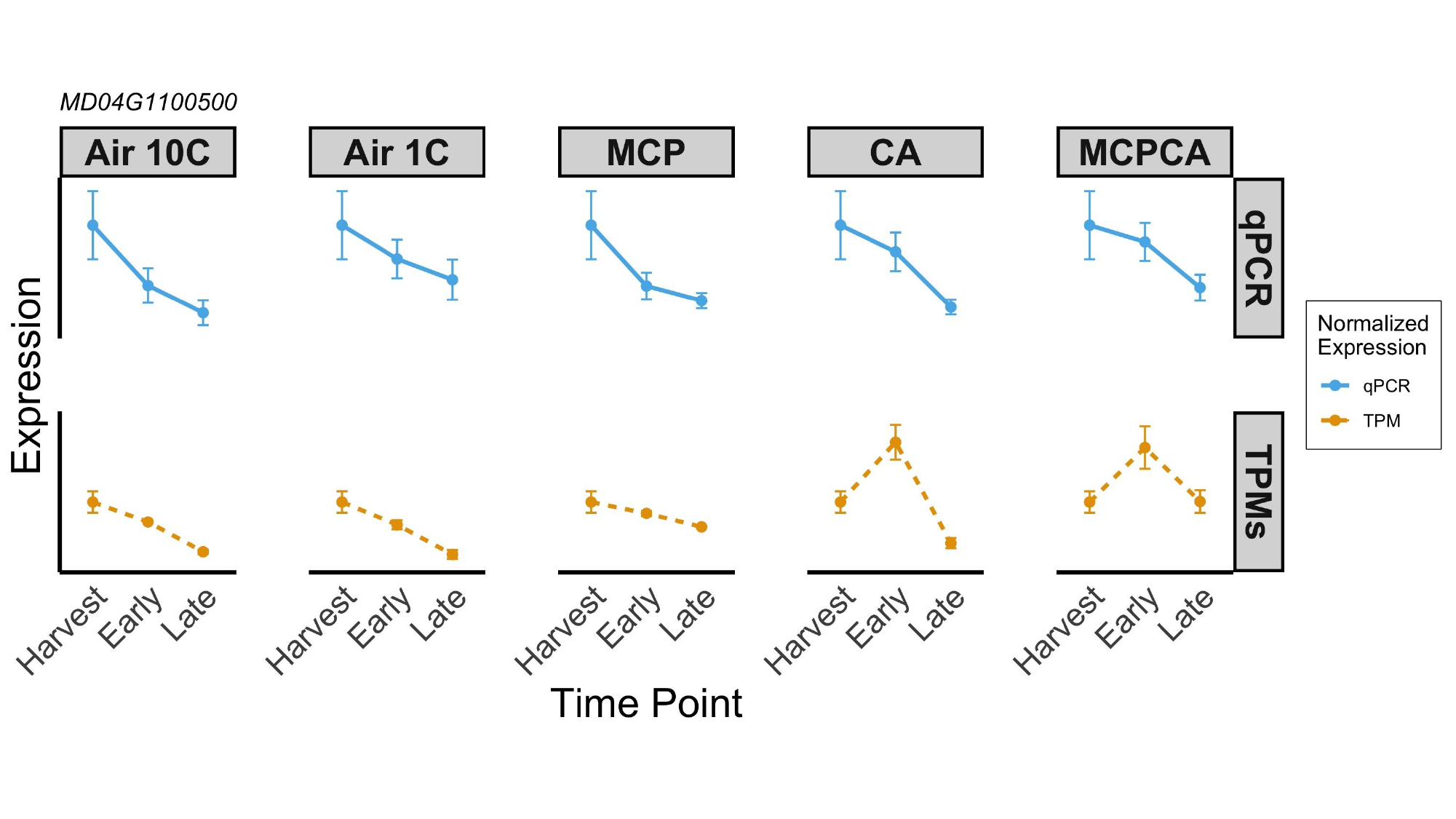

## Slide 9
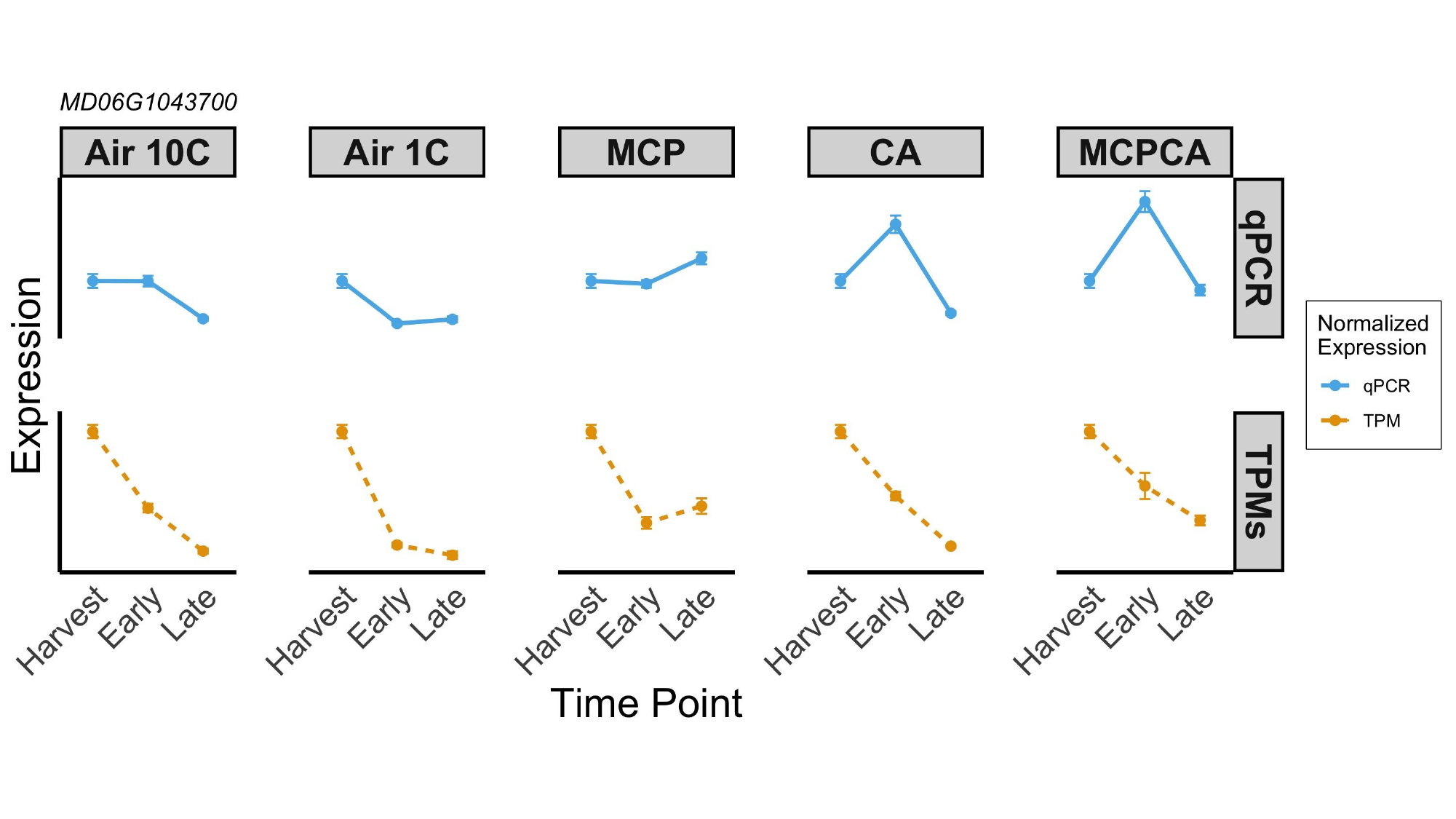

## Slide 10
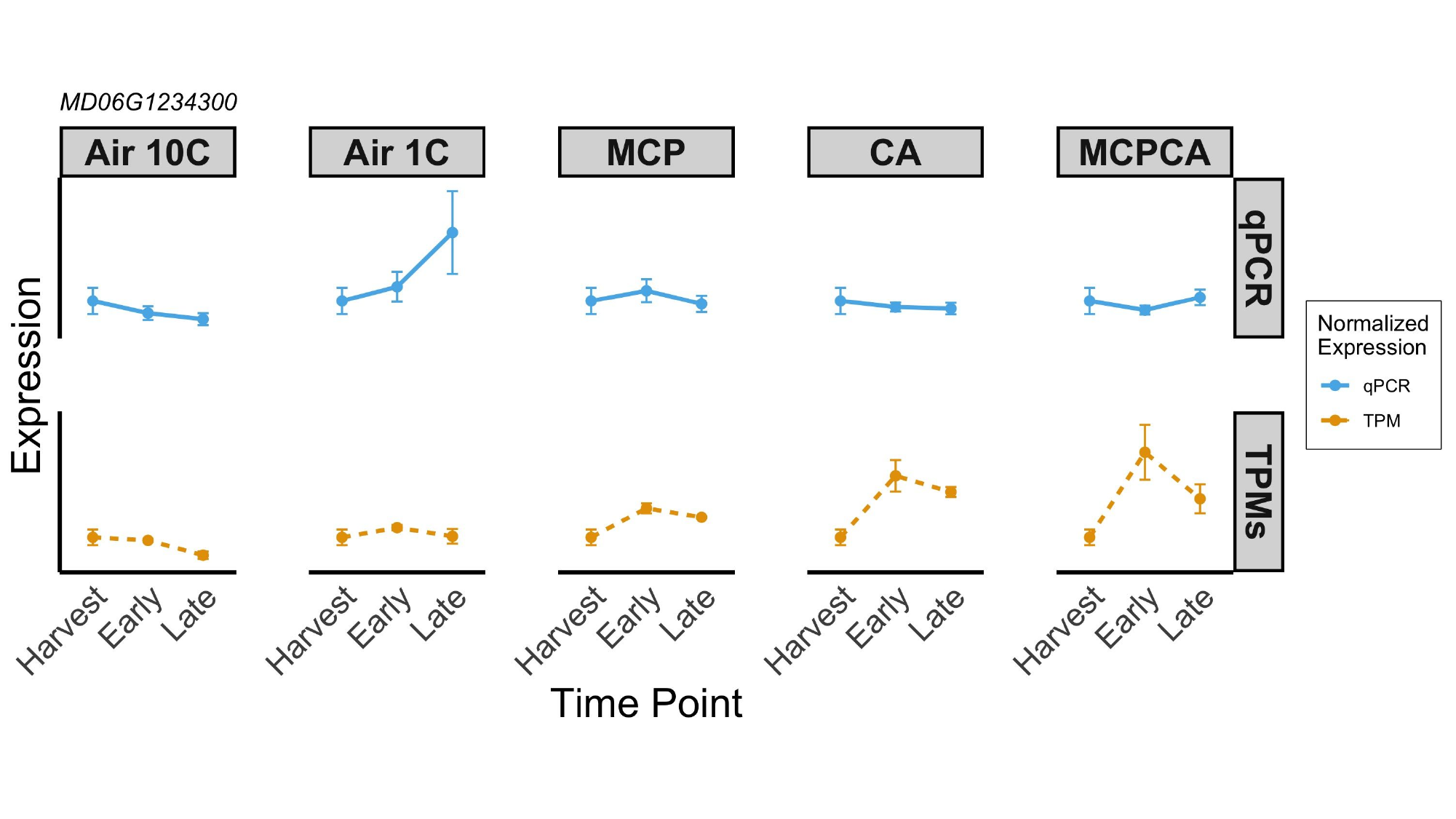

## Slide 11
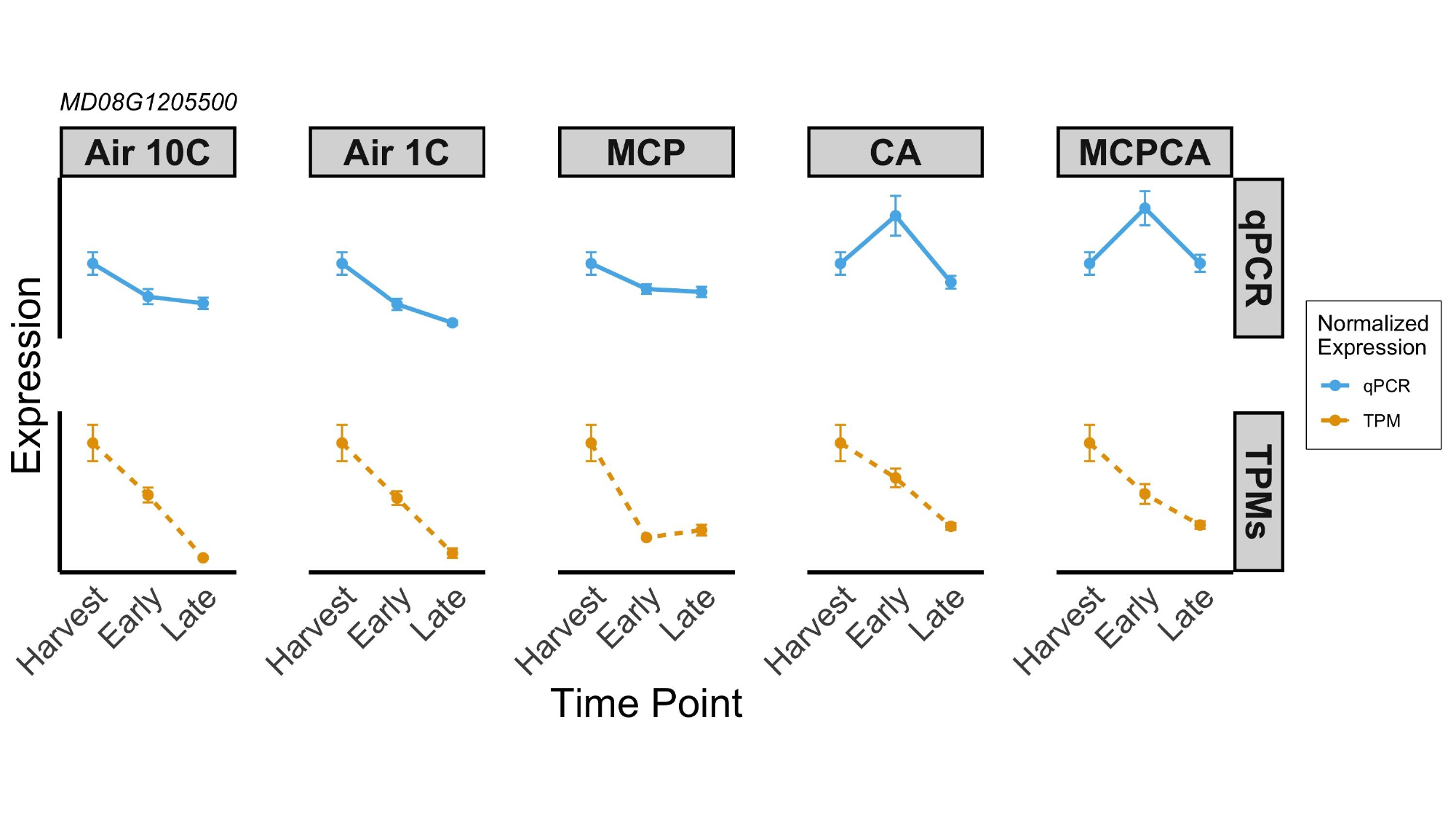

## Slide 12
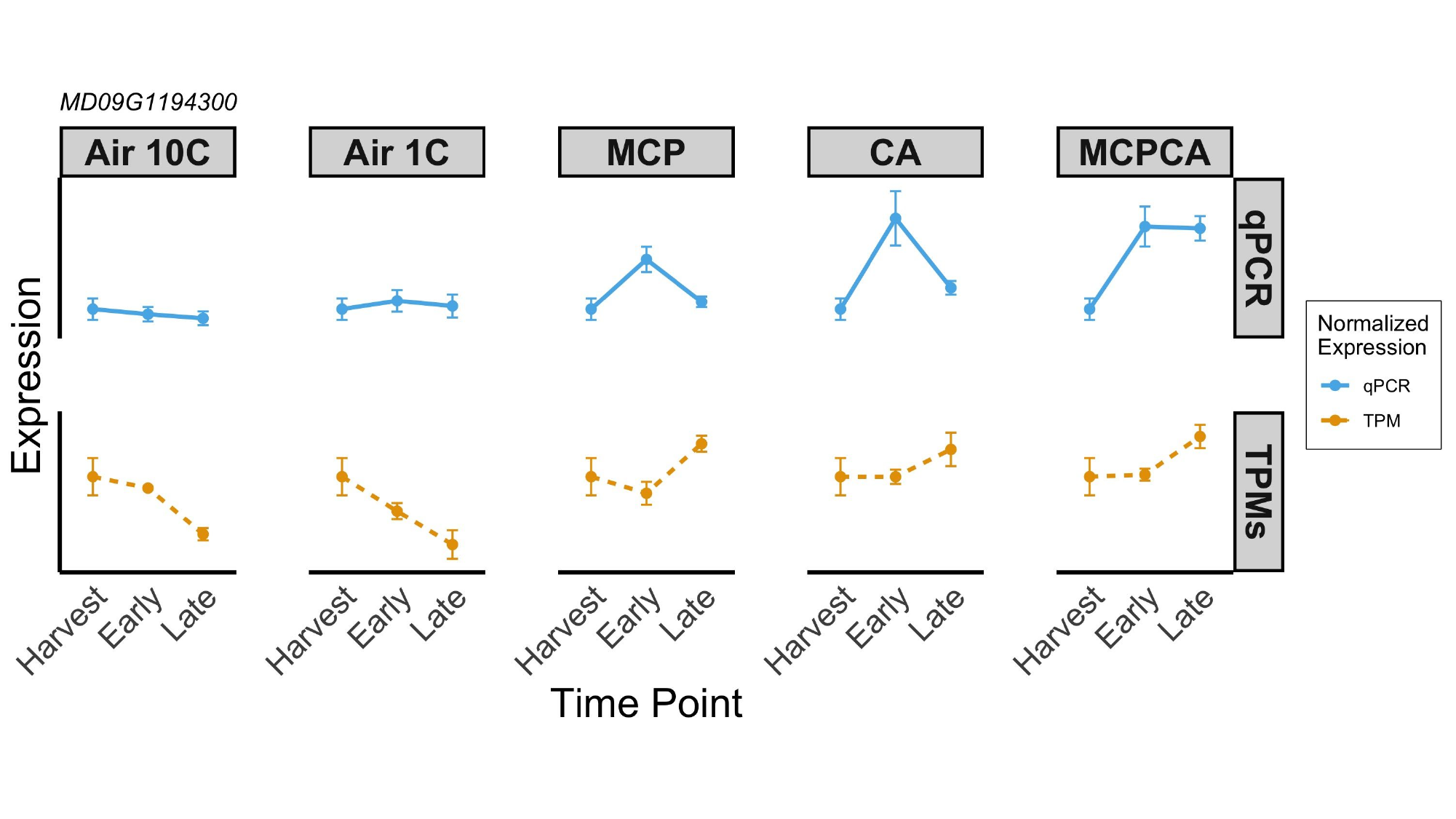

## Slide 13
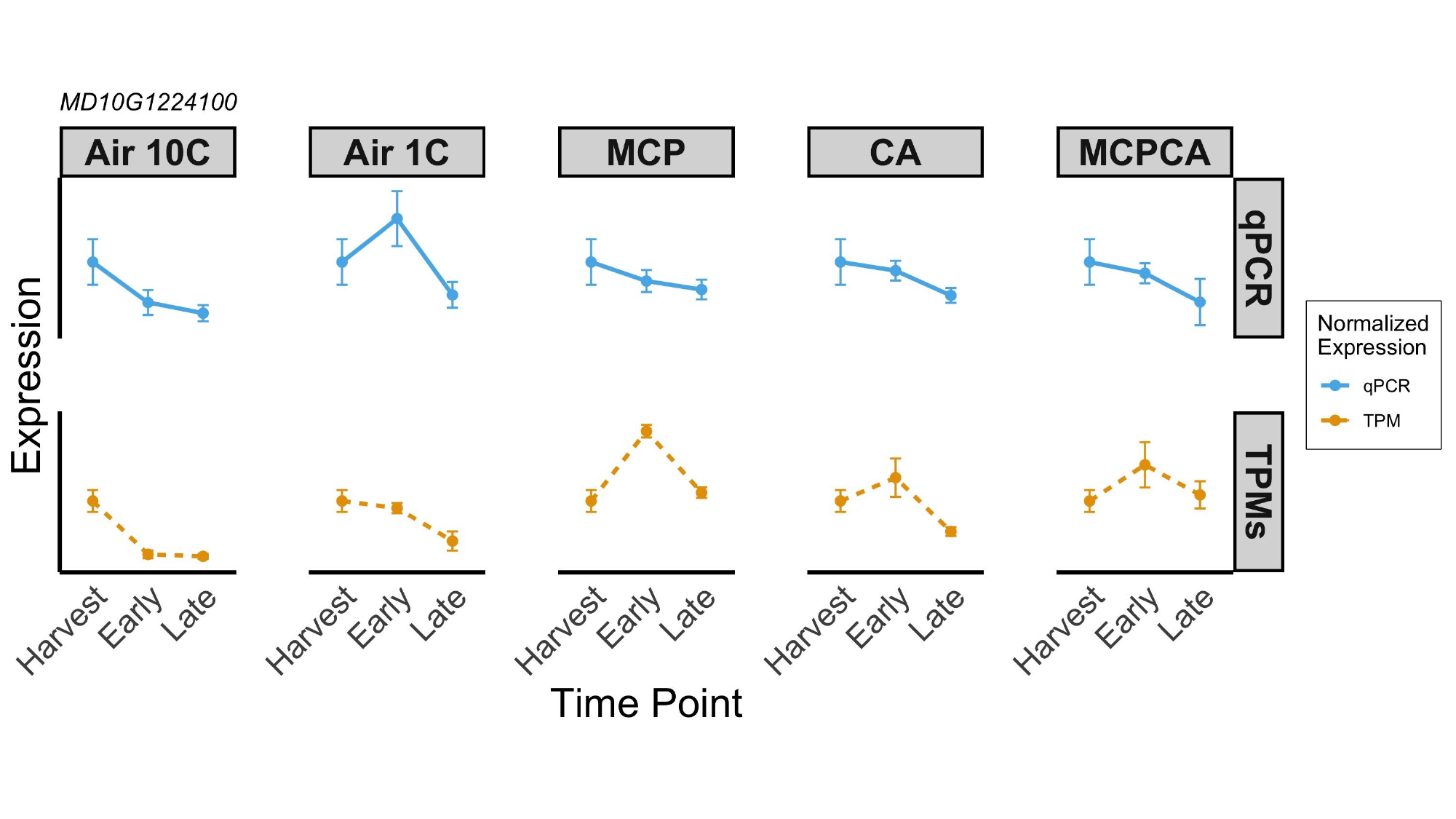

## Slide 14
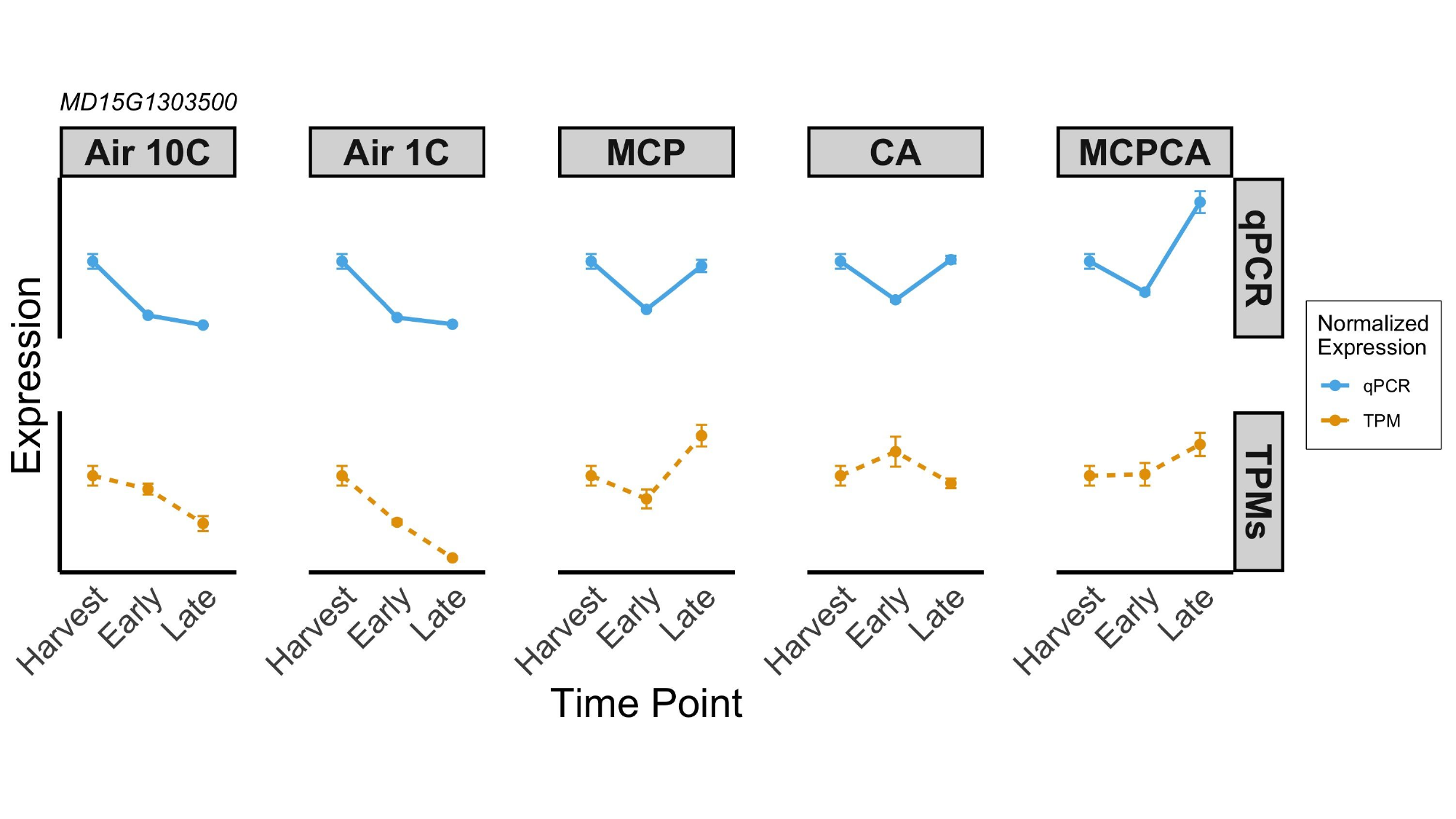

## Slide 15
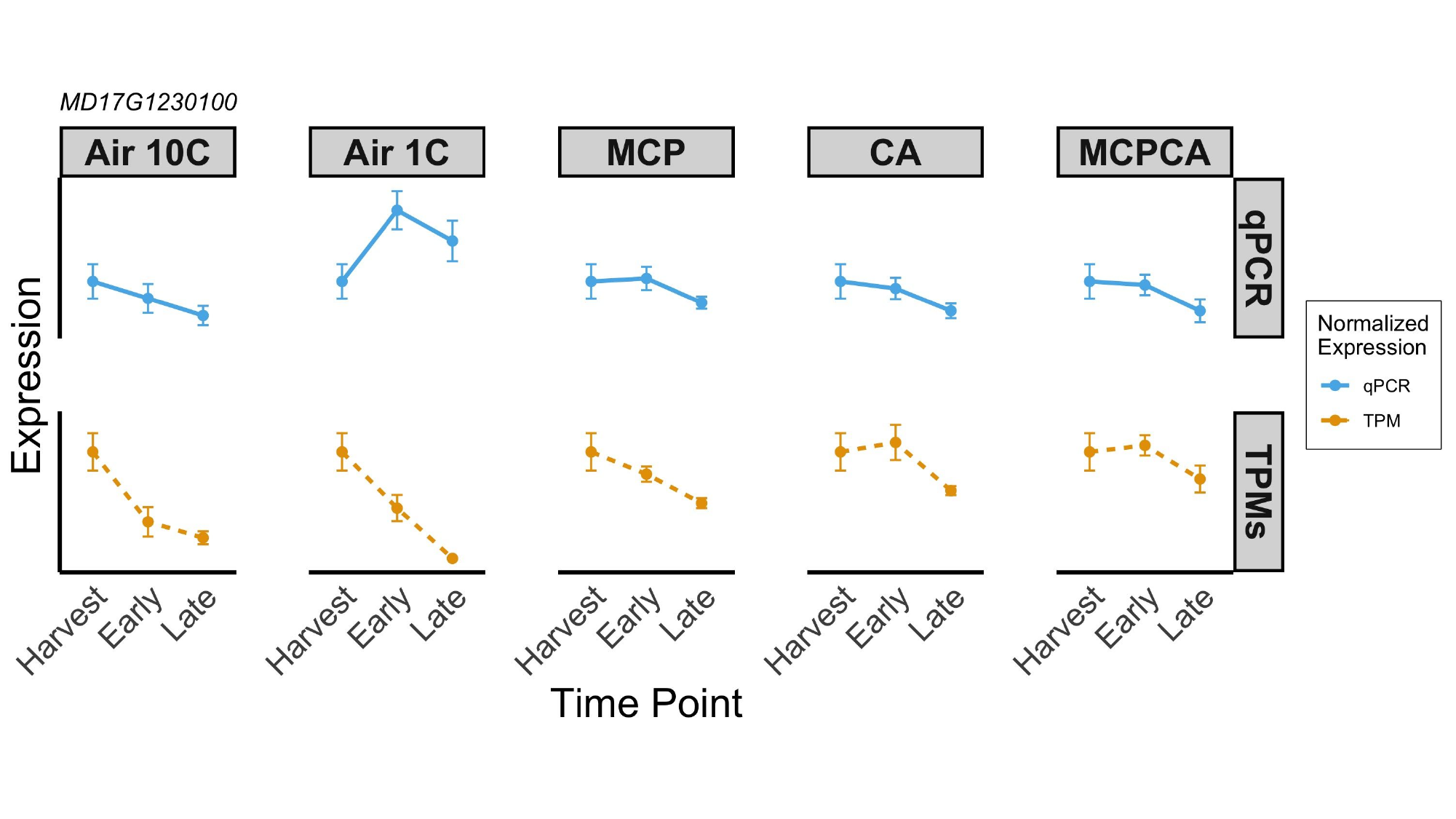

Supplement: S3 File — Compressed series of plots showing the difference between qPCR and RNA-seq patterns of the selected 15 genes. (ZIP) [file pone.0297015.s003.zip › Supplemental_File_3-qPCRvsRNASeq-PatternComparison/ExpressionPatterns_ByGOI_qPCRvsRNASeq.pptx]

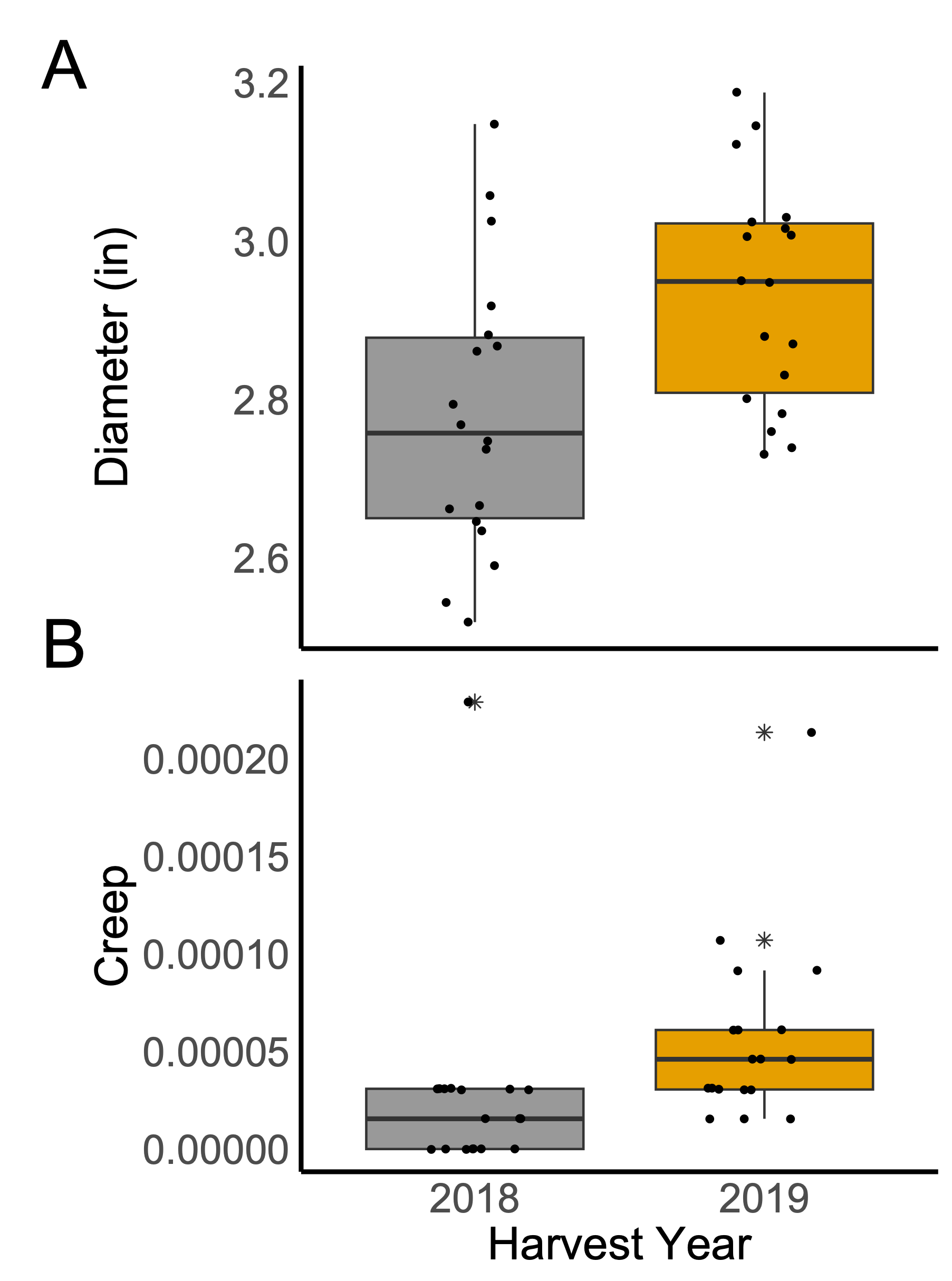

Supplement: S1 Fig — A) Differences in fruit diameter at harvest between 2018 and 2019. B) Differences in creep at harvest during the same period. (PNG) [file pone.0297015.s005.png]

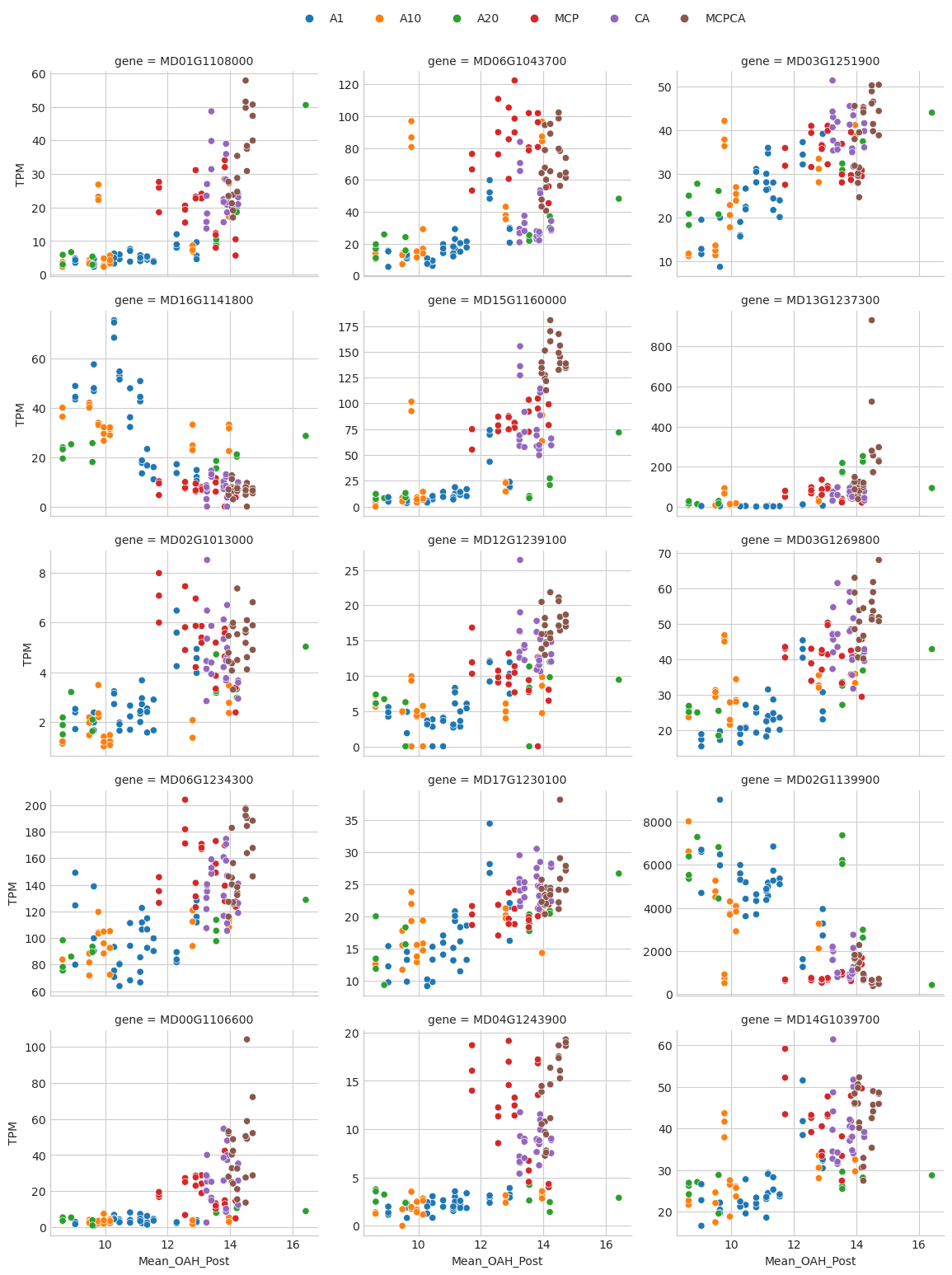

Supplement: S2 Fig — The expression level (y-axis) of genes using TPM normalized counts over time (x-axis) for the top 15 genes identified using the random forest model. (PNG) [file pone.0297015.s006.png]

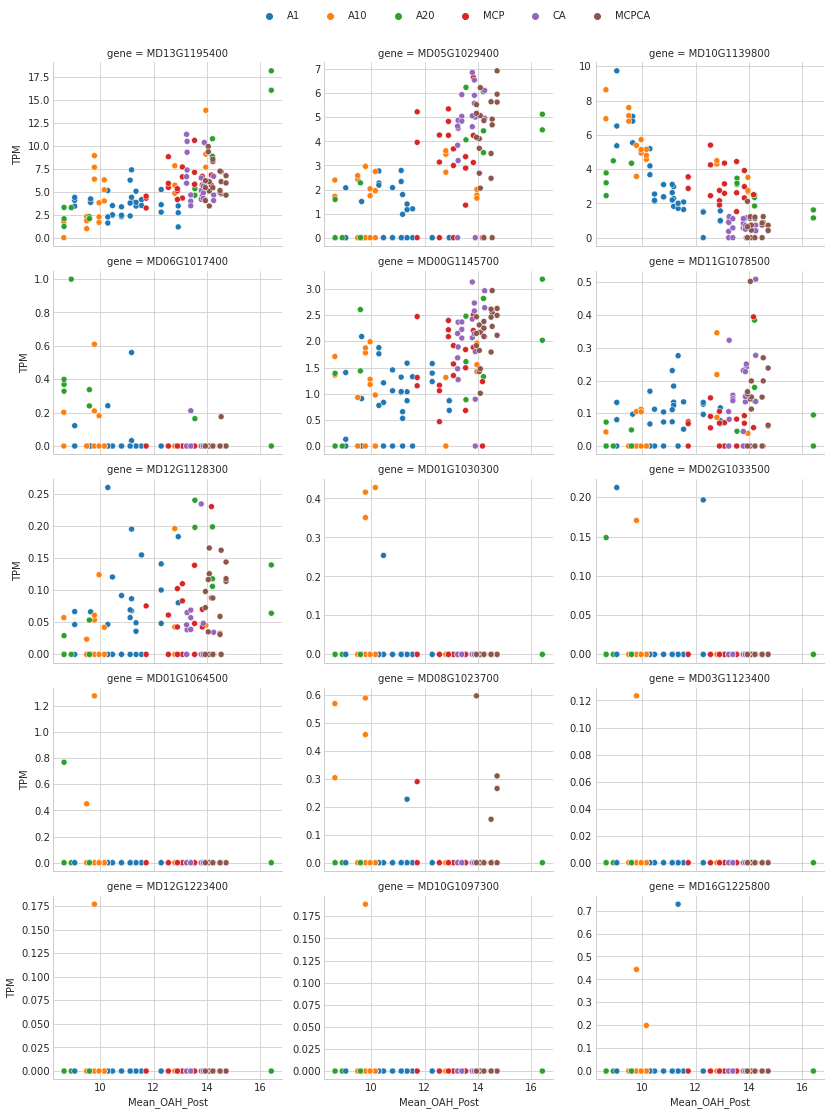

Supplement: S3 Fig — The expression level (y-axis) of genes using TPM normalized counts over time (x-axis) for the top 15 genes identified using the elastic net model. (PNG) [file pone.0297015.s007.png]

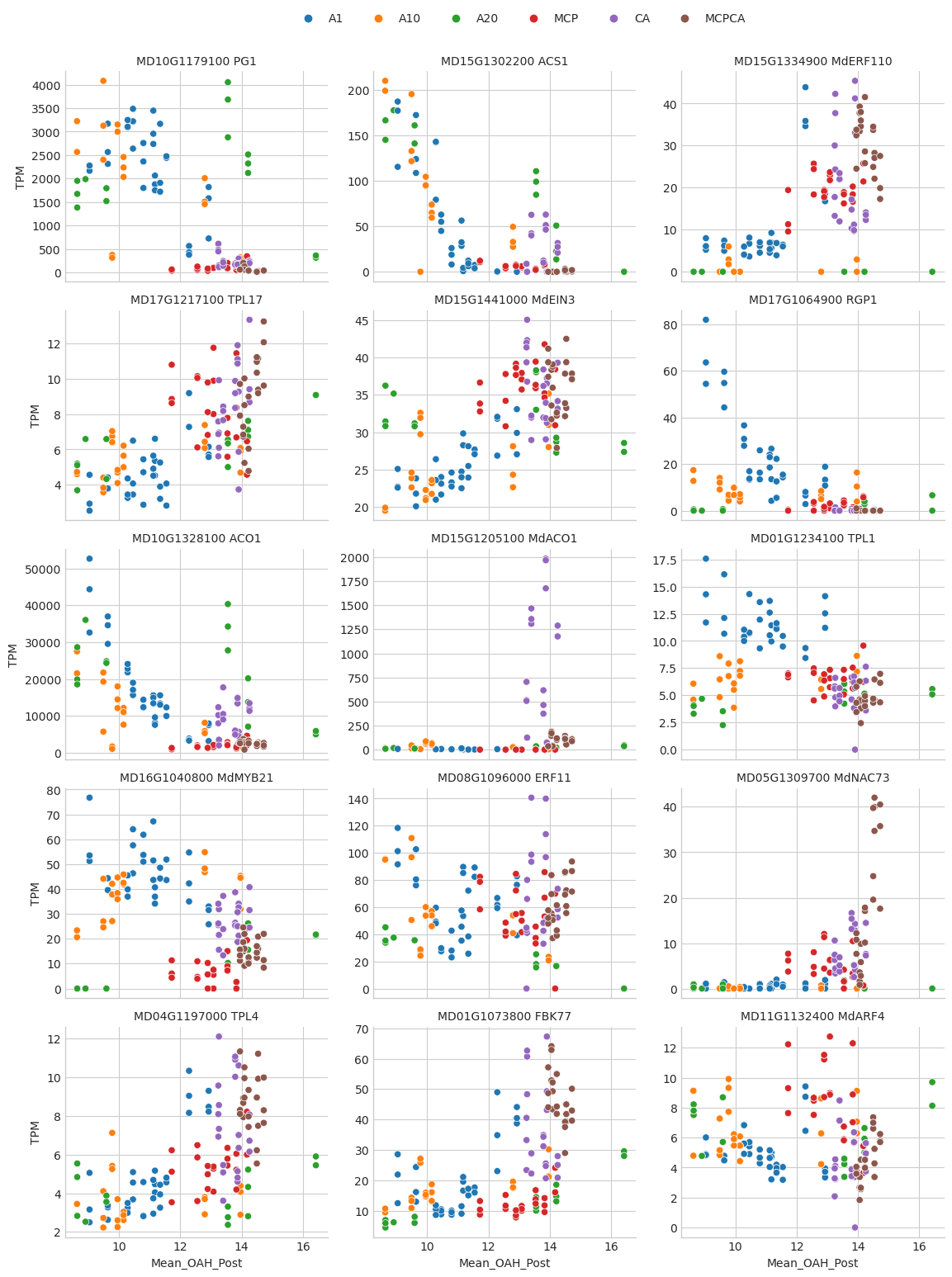

Supplement: S4 Fig — The expression level (y-axis) of genes using TPM normalized counts over time (x-axis) for the top 15 genes identified from the literature. (PNG) [file pone.0297015.s008.png]

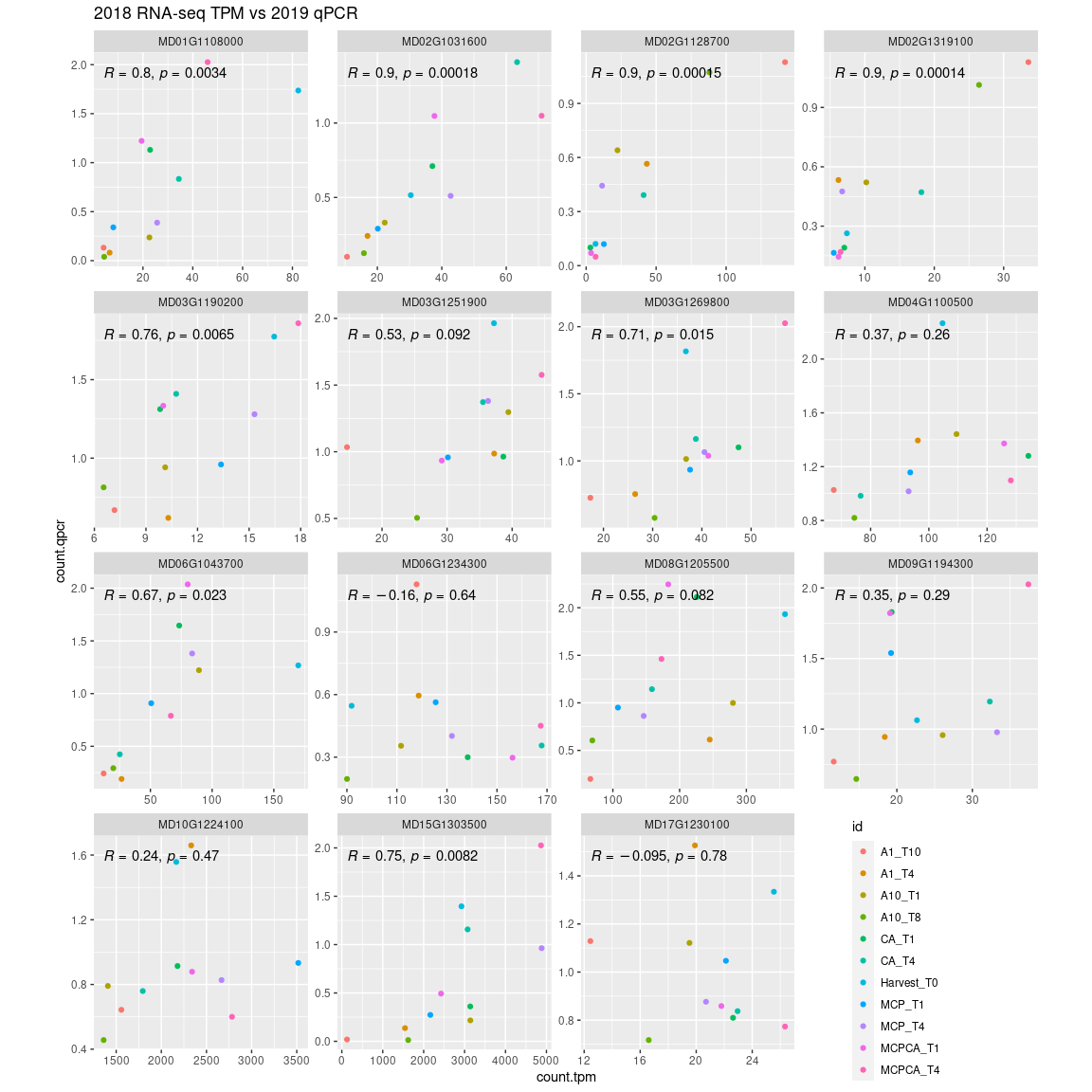

Supplement: S5 Fig — Scatterplot of gene expression (y-axis) using TPM normalized counts vs qPCR levels (x-axis). (PNG) [file pone.0297015.s009.png]
